# Dendritic Iron(III) Carbazole Complexes: Structural, Optical, and Magnetic Characteristics

**DOI:** 10.3390/ma14185445

**Published:** 2021-09-20

**Authors:** Matvey Gruzdev, Ulyana Chervonova, Arkadiy Kolker, Nadezhda Fomina, Ekaterina Zueva, Valerya Vorobeva, Denis Starichenko, Alexander Korolev

**Affiliations:** 1 G.A. Krestov Institute of Solution Chemistry of Russian Academy of Sciences, 153045 Ivanovo, Russia; uch@isc-ras.ru (U.C.); amk@isc-ras.ru (A.K.); nafnafnaf74@mail.ru (N.F.); 2 Department of Inorganic Chemistry, Kazan National Research Technological University, 420015 Kazan, Russia; zueva_ekaterina@mail.ru; 3 Zavoisky Physical-Technical Institute, FRC Kazan Scientific Center of RAS, 420029 Kazan, Russia; vvalerika@gmail.com; 4 M.N. Mikheev Institute of Metal Physics of the Ural Branch of the Russian Academy of Sciences, 620108 Ekaterinburg, Russia; starichenko@imp.uran.ru (D.S.); korolyov@imp.uran.ru (A.K.)

**Keywords:** iron(III) complexes, carbazole, fluorescence, HS and LS spin state, spin crossover, magnetic susceptibility, antiferromagnetic interactions

## Abstract

This paper focuses on the synthesis, structural characterization, and study of the optical, magnetic, and thermal properties of novel architectures combining metal ions as magnetoactive centers and photoactive blocks formed by carbazole units. For this purpose, a series of azomethine complexes of the composition [Fe(L)_2_]X (L = 3,6-bis[(3′,6′-di-*tert*-butyl-9-carbazol)-9-carbazol]benzoyloxy-4-salicylidene-N′-ethyl-N-ethylenediamine, X = NO_3_^−^, Cl^−^, PF_6_^−^) were synthesized by the reaction of metal salts with Schiff bases in a mixture of solvents. The UV–Vis absorption properties were studied in dichloromethane and rationalized via time-dependent density functional theory (DFT) calculations. Upon excitation at 350 nm, the compounds exhibited an intense dual fluorescence with two emission bands centered at ~445 and ~485 nm, which were assigned to *π*_carb_–π* intraligand and *π*_carb_–d_Fe_ ligand-to-metal charge-transfer excited states. EPR spectroscopy and SQUID magnetometry revealed solid-state partial spin crossover in some compounds, and antiferromagnetic interactions between the neighboring Fe(III) ions.

## 1. Introduction

The possibility of combining various structural units into one molecule to obtain new properties required for technological applications is of undoubted interest to chemists. Examples of multifunctional systems include dendrimers—hyperbranched macromolecules with a well-defined molecular structure [1,2]. The design and synthesis of π–π conjugated dendrimers attract permanent attention because of their widespread use as organic light-emitting diodes, electroluminescent devices, solar cells, and organic field-effect transistors [3,4]. An example of the most promising chromophores for creating such systems is carbazole, along with its derivatives [5,6]. This is a group of nitrogen-containing aromatic compounds with useful electronic properties and a π-conjugated system capable of charge transfer, as well as easily undergoing chemical modifications due to the introduction of various functional groups into a structurally rigid carbazole ring [7]. The inclusion of metal ions in the dendrimers’ cores gives the system new unusual properties, including the ability to shield excited states from quenching processes, collect sunlight in the dendrimers’ cores, and convert incident ultraviolet light into visible or infrared radiation [8]. In this regard, emission complexes of transition metals—and especially iron(III) complexes, in which the metal center can adopt two different spin states (low-spin (LS) and high-spin (HS)) interconverted by external influences (e.g., light irradiation, temperature, pressure, nature of the solvent [9,10])—can find extensive potential application. These properties explain the increased interest in the design of metal dendrimers based on carbazole by means of coordinated self-assembly [11]. Analysis of the available papers on carbazole metal dendrimers shows that the main research areas dealing with the physical properties of these systems are devoted to catalysis [12], light-absorbing and luminescent properties [13], electrochemical behavior [14], sensory properties [15], and medicines [16]. The magnetic properties of iron-containing dendrimers—and especially a combination of magnetic properties with others in one material, their mutual influence, and mutual control—have not been studied thus far. Most previous publications on this topic are devoted to spin-crossover (SCO) iron(II) compounds (*S* = 0 ÷ 2), while iron(III) complexes have a number of advantages (e.g., structural, magnetic, photomagnetic, and relaxation).

Thus, the aim of this work is to synthesize a series of new iron(III) complexes with a branched Schiff base of carbazole derivatives, and explore their structural, optical, and magnetic properties in solution and solid state via a wide range of experimental techniques complemented by quantum chemical calculations and theoretical analysis.

## 2. Materials and Methods

### 2.1. Materials

The commercially available reagents and solvents employed in this study were of chemically pure grade, and were used without further purification unless otherwise stated. [3,6-Bis(3′,6′-di-*tert*-butyl-9′H-carbazol)-9H-carbazol]benzoyloxy-2-hydroxy benzaldehyde was obtained via the reaction of 3,6-di[3′,6′-bis-*tert*-butyl-9′H-carbazol]-9H-carbazol-9-yl-4-benzoic acid with 2,4-dihydroxybenzaldehyde in CH_2_Cl_2_ solution in the presence of DCC and DPTS as a catalyst (Steglich esterification) [17].

### 2.2. Physical Measurements

Infrared (IR) spectra were recorded on a Vertex 80V (Bruker, Ettlingen, Germany) device in the regions of 7500–370 cm^–1^ and 670–190 cm^–1^ from pellets with KBr and CsBr. The ^1^H NMR spectral studies (500.17 MHz) were performed on a Avance-500 instrument (Bruker, Ettlingen, Germany) in CDCl_3_, relative to TMS as an external reference. Mass spectra were recorded using the MALDI-ToF method on a Ultraflex III Bruker mass spectrometer (Bruker, Ettlingen, Germany) in the positive ion mode (matrix are 2,5-dihydroxybenzoic acid and sinapinic acid). Thermogravimetric analysis (TGA) was performed using a NETZSCH TG 209 F1 analyzer (NETZSCH, Selb, Germany) by the onset of the gravimetric reduction point of the TG curve. DSC measurements were carried out using DSC 204 F1 Phoenix (NETZSCH, Selb, Germany) with a *μ*-sensor. High-temperature DSC was performed using a synchronous thermal analyzer (DSC/DTA/TG) (NETZSCH Geraetebau GmbH, Selb, Germany) with a skimmer mass spectrometric system for analyzing the vapor phase. The sample placed into a platinum crucible was heated in argon flow at a rate of 10 K/min. The scan rate at heating and cooling was 10 K/min in an argon atmosphere. The phase transition behavior was observed by means of an Altami Polar 3 polarizing microscope (Altami, Saint-Petersburg, Russia) equipped with a hot stage HS 82 (Mettler Toledo, Schwerzenbach, Switzerland). The transition temperatures were determined with a scan rate from 2 to 10 K/min using a temperature controller (Mettler Toledo, HS 1). Absorption and fluorescence spectra were recorded on an CM 2203 SOLAR (JSC SOLAR, Minsk, Belarus) spectral fluorimeter at room temperature in solution (CH_2_Cl_2_). The fluorescence quantum yield was determined relative to the quinine bisulfate solution as a standard (*ϕ*_st_ = 0.55) [18]. Elemental analyses of crystalline compounds were carried out on a FlashEA 1112 to C, H, N, O (Thermo Fisher Scientific Inc., Waltham, MA, USA). X-ray powder diffraction analysis was carried out on a D2 PHASER diffractometer (Bruker, Ettlingen, Germany). In all cases, filtered emission copper (Cu) K_α_ (λ = 1.5406 Å) with a step of 0.0200 was used. EPR experiments were carried out on polycrystalline (powder) samples using an X-band (9.41 GHz) CW-EPR EMXplus Bruker spectrometer (Bruker, Ettlingen, Germany) equipped with helium ER 4112HV and digital ER 4131VT temperature control systems (Bruker, Ettlingen, Germany). Magnetic measurements were carried out using a Quantum Design MPMS-5XL SQUID magnetometer (Quantum Design, San Diego, CA, USA). The temperature dependences of the static magnetic susceptibility, *χ*(*T*), were measured on polycrystalline (powder) samples within the magnetic field *H* = 10 kOe under heating and cooling regimes in the range of 2.0–300 K. The magnetization curves, *M*(*H*), were obtained at 2.0 K within the field range of 0–50 kOe. The diamagnetic contribution of the organic framework to the total SQUID signal was temperature-independent and was taken into account by subtraction of the respective Pascal constants.

### 2.3. DFT Calculations

All quantum chemical calculations were performed using algorithms implemented in the ORCA package (version 4.0) (Max Planck Institute fuer Kohlenforschung, Mülheim an der Ruhr, Germany) [19]. The RIJCOSX approximation for the two-electron integrals was used throughout. Full geometric optimizations were carried out using the B3LYP/LANL2DZ computational procedure, which employs the B3LYP functional [20,21] in conjunction with the LANL2DZ basis set [22] and associated ECP [23,24,25] for Fe. The vertical electronic transitions (absorption spectra) were calculated via time-dependent DFT (CAM-B3LYP/LANL2DZ) using the Tamm–Dancoff approximation [26,27,28]. According to our test calculations, the incorporation of bulk solvation effects (solvent–dichloromethane) through the C-PCM model [29] does not lead to significant changes in the calculated spectra, and so no further attempt was made to include solvation interactions in the computational procedure. The calculated excitations were interpreted by analyzing the so-called natural transition orbitals (NTOs) for each excited state. The occupations of NTO pairs reflect the contribution of each NTO pair to a given electronic transition.

### 2.4. Synthesis: General Procedure

**Scheme 1:**
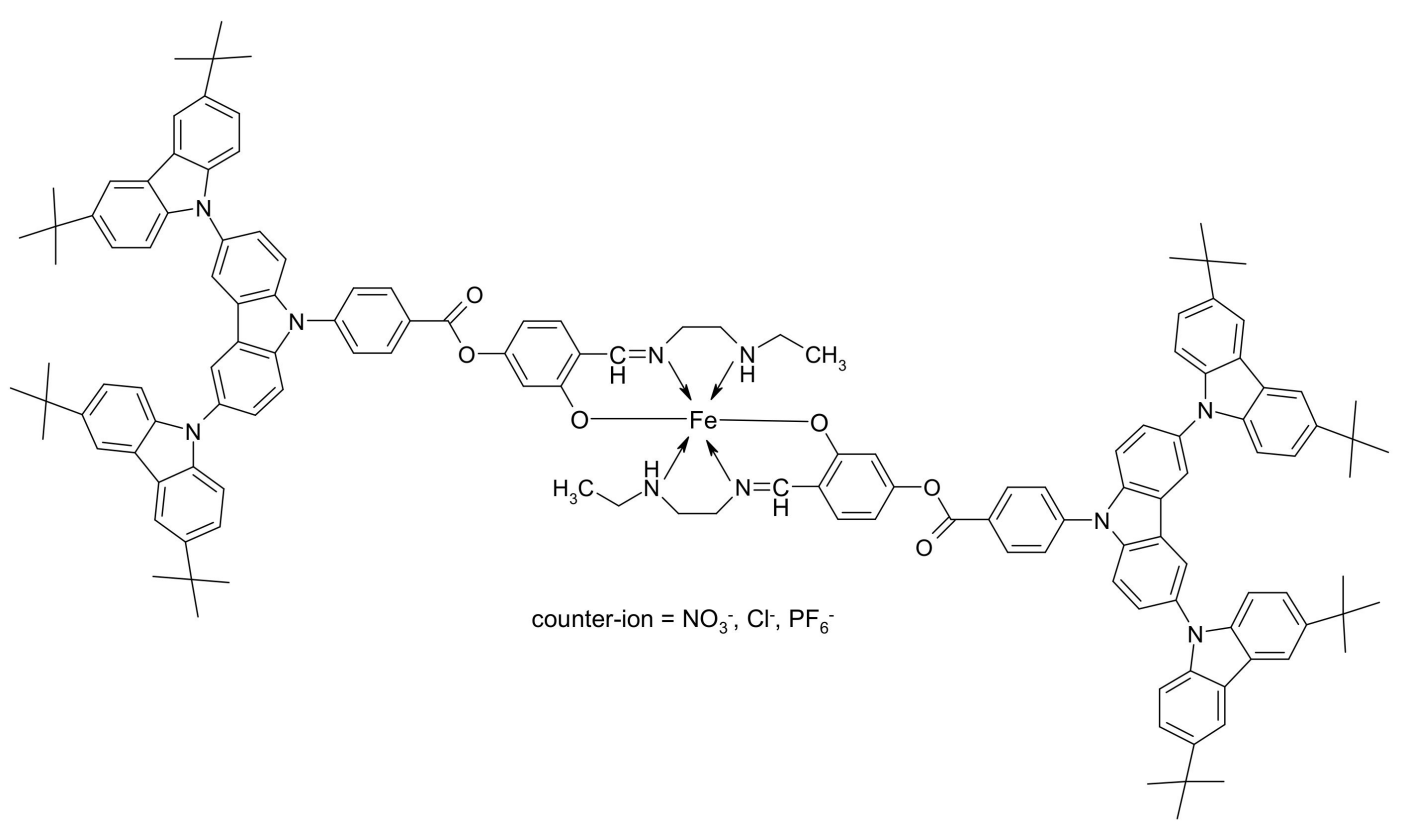
Structural formula of complexes **1–3**.

A weighted portion of [3,6-bis(3′,6′-di-tert-butyl-9′H-carbazol)-9H-carbazol]benzoyloxy-2-hydroxybenzaldehyde (1 mol) was dissolved in C_6_H_6_ under heating and mixed with *N*′-ethyl-*N*-ethyleneamine (1 mol) dissolved in EtOH. The mixture was stirred for 10 min, and a weighted portion of KOH (in excess of 1 mmol) in EtOH was added. Then, an alcohol solution of the metal salts Fe(NO_3_)_3_·9H_2_O (0.5 mol), FeCl_3_ (0.5 mol), or Fe(NO_3_)_3_·9H_2_O (0.5 mol)/KPF_6_ (1 mol) was slowly added (drop by drop). After 3 h, the reaction mass was cooled for 12 h. The formed precipitate was filtered off, washed with ethanol, and freeze-dried (Scheme 1).

- *[Fe(L)_2_]NO_3_* (**1**). Product is a light-brown fine-dispersed powder. Yield is 85.78% (0.39 g). FT-IR (KBr, ν, cm^–1^): 3424 (m, NH), 3045 (m, Ph–H), 2960–2865 (s, CH_2_, CH_3_), 1722 (s, –COO–), 1607 (s, HC=N), 1388 (s, NO_3_^−^). Anal.: Calculated (%): C, 77.08; H, 6.65; N, 7.07; O, 6.11; Fe, 2.59. C_140_H_144_N_10_O_6_Fe·NO_3_. Found (%): C, 77.16; H, 6.69; N, 7.02; O, 6.59; Fe, 2.54. MS (*m/z*): Calculated 1032.84; Found 1034.45 [L]^+^.
- *[Fe(L)_2_]Cl* (**2**). Product is a grey fine-dispersed powder. Yield is 70.61% (0.32 g). FT-IR (KBr, ν, cm^–1^): 3421 (m, NH), 3044 (m, Ph–H), 2959–2864 (s, CH_2_, CH_3_), 1720 (s, –COO–), 1609 (s, HC=N). Anal.: Calculated (%): C, 78.03; H, 6.74; N, 6.5; O, 4.45; Fe, 2.63; Cl, 1.65. C_140_H_144_N_10_O_6_Fe·Cl. Found (%): C, 77.98; H, 6.78; N, 6.43; O, 4.67; Fe, 2.56; Cl, 1.58. MS (*m/z*): Calculated 1032.84; Found 1034.81 [L]^+^.
- *[Fe(L)_2_]PF_6_* (**3**). Product is a grey fine-dispersed powder. Yield is 70.83% (0.34 g). FT-IR (KBr, ν, cm^–1^): 3419 (m, NH), 3050 (s, Ph–H), 2960–2865 (s, CH_2_, CH_3_), 1721 (s, –COO–), 1605 (s, HC=N), 839 (m, PF_6_^−^), 559 (m, PF_6_^−^). Anal.: Calculated (%): C, 74.26; H, 6.41; N, 6.18; O, 4.24; Fe, 2.51; P, 1.37; F, 5.03. C_140_H_144_N_10_O_6_Fe·PF_6_. Found (%): C, 74.28; H, 6.44; N, 6.08; O, 4.11; Fe, 2.63; P, 1.48; F, 4.98. MS (*m/z*): Calculated 1032.84; Found 1034.16 [L]^+^.

All spectra are given in Figures S1–S10.

## 3. Results

### 3.1. Infrared Spectroscopy

The FTIR spectra of complexes **1**–**3** were measured in the ranges of 7500–370 cm^–1^ and 670–190 cm^–1^ from KBr (Figures S2–S4) and CsBr (Figures S5–S7) pellets. The absorption band at 3420 cm^–1^ can be assigned to the stretching vibrations of the NH_2_ group [30]. This can be explained by the fact that water molecules were removed via lyophilization of the samples, and KBr was additionally calcined before pellet preparation. The FTIR spectra showed strong absorption bands at 1720–1722 cm^–1^ and 1605–1609 cm^–1^, attributed to the frequencies of the stretching vibrations of carbonyl (COO) and imine (C=N) [31], respectively. Counterions give the following absorption bands in the spectrum: PF_6_^−^—832 and 558 (broad) cm^–1^ [30,32]; NO_3_^−^—1385 cm^–1^. The involvement of phenolic oxygen in the formation of the coordination site is confirmed by the stretching vibrations of the Fe–O bond at 423 cm^–1^. For complex **3**, the vibrations of the Fe–N bond in the region of 560 cm^–1^ are overlapped with a broadened intense signal from the PF_6_^−^ counterion. Absorption bands at 275 and 330 cm^–1^ can be attributed to the Fe–N bond vibrations [33,34,35].

### 3.2. ^1^H NMR Spectroscopy

Since the complexes contain a paramagnetic metal ion in their structure, the ^1^H NMR spectra with broadened low-intensity signals were recorded (Figures S8–S10). The participation of NH groups in complexation with Fe(III) ions gives rise to a new signal with a chemical shift of 4.48 ppm. At the same time, the COH (11.30 ppm) and OH (9.89 ppm) protons of the aldehyde molecule participate in the formation of the salicylidene CH=N fragment and the Fe–O bond, respectively; therefore, their signals are not observed in the spectra of the complexes. This fact is also confirmed by the IR spectroscopic data.

### 3.3. Thermal Analysis

**Figure 1:**
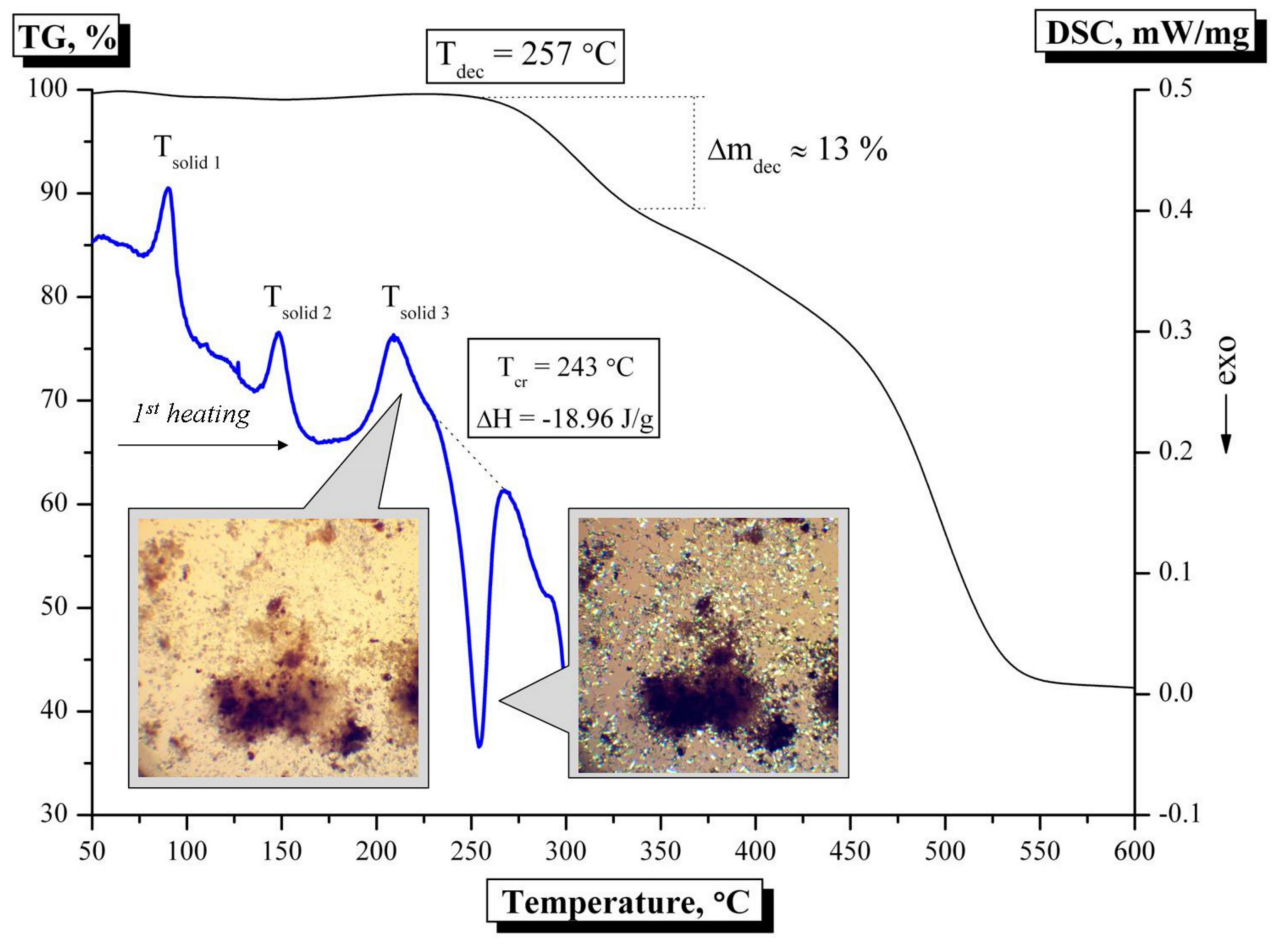
TG and DSC curves (heating cycle), and photographs of complex **3**.

Information about the thermal properties of compounds **1**–**3** was obtained via the TG/DTG and DSC techniques (Figure 1, Figures S11 and S12). The nature of phase transitions is determined by the nature of the counterion. Compound **1** is characterized by “solid–solid” type phase transitions reproducible in heating and cooling cycles at 134–140 °C. When the counterion is replaced by metathesis by the PF_6_^−^ ion (**3**), the system undergoes a series of solid-state transitions at *T*_solid 1_ = 83 °C, *T*_solid 2_ = 140 °C, and *T*_solid 3_ = 197 °C (Figure 1). Compound **2,** synthesized from the iron(III) chloride salt, exhibits a different behavior. No crystalline transitions were detected by DSC measurements; only the amorphous state of the sample, with a glass transition temperature of 143 °C, was found. Within the whole series of compounds, no melting or softening point was observed. It was found that all of the compounds have high thermal stability up to 250 °C (373 °C for **1**, 390 °C for **2**, and 257 °C for **3**). A process with an exo-effect occurs near the decomposition temperature, which can be interpreted as crystallization of a substance under the influence of temperature. This is confirmed by the polarizing optical microscopy (POM) data collected upon heating the sample (Figure 1). It was found that the temperature of crystallization process depends on the nature of the counterion (NO_3_^−^: 319 °C; Cl^−^: 386 °C; PF_6_^−^: 243 °C). This is probably due to the processes of supramolecular aggregation, which occurs with the formation of intermolecular hydrogen bonds.

### 3.4. Optical Properties

**Figure 2:**
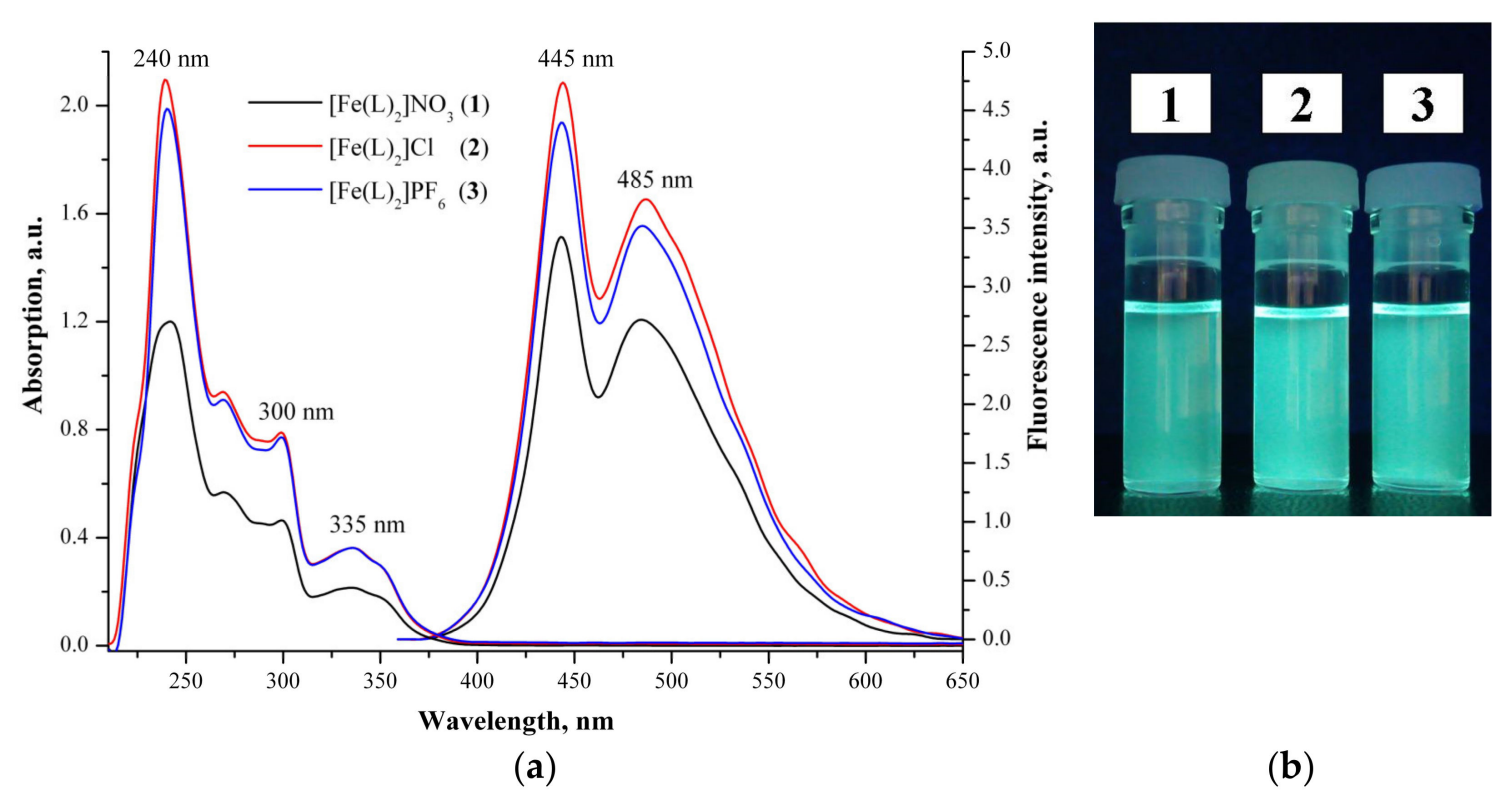
The absorption and fluorescence spectra of compounds **1–3** in dichloromethane, 5 × 10^−6^ mol/L, *λ*_exc_ = 350 nm, *T* = 298.15 K (**a**), and their photographic images in CH_2_Cl_2_ solution (**b**).

The photophysical properties of compounds **1**–**3** were studied via UV–Vis and fluorescence spectroscopy in dichloromethane. The corresponding spectra are shown in Figure 2. The absorption profiles are similar for all compounds, and mimic the behavior of a previously studied [36] complex containing the same magnetoactive core and two 3,6-di-*tert*-butyl-carbazole moieties on the ligand periphery. An intense absorption band in the UV region (~240 nm) is characteristic of intraligand (IL) transitions. Two additional absorption bands centered at ~300 and ~335 nm could be assigned to ligand-to-metal (LMCT), metal-to-metal (MMCT), and intraligand (ILCT) charge transfers, as well as to intraligand (IL) transitions. Upon excitation at 350 nm, the compounds exhibit an intense dual fluorescence with two emission bands centered at ~445 and ~485 nm. Thus, the increase in the number of carbazole-based photoactive fragments in the molecular structure of the complex results in a double-band emission (with maxima at 445 and 485 nm), in contrast to the single emission pattern (with a maximum at 449 nm) observed for a complex functionalized by only two carbazole moieties (*λ*_exc_ = 345 nm) [36].

The fluorescence quantum yield value was estimated relative to the quinine bisulfate solution as a standard (*ϕ*_st_ = 0.55), and reached 37.5, 45.6, and 37.1% for complexes **1**, **2**, and **3**, respectively.

### 3.5. TD-DFT Calculations

**Figure 3:**
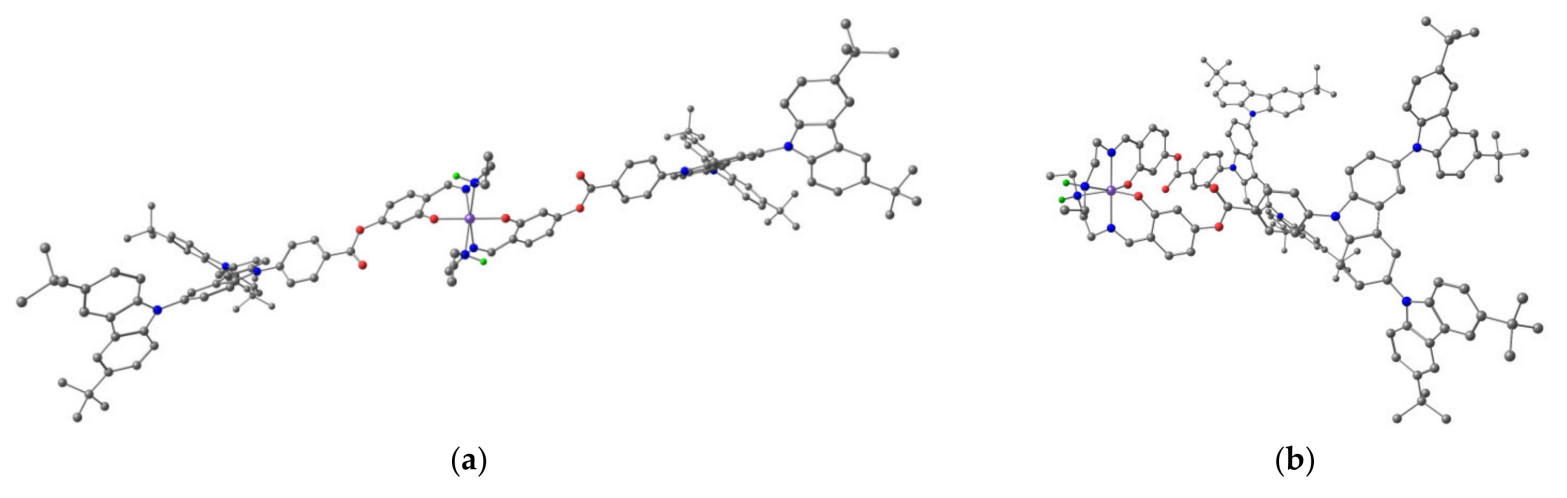
The DFT-optimized structures of the *fac* (**a**) and *mer* (**b**) isomers of the cationic complex. Only amine hydrogen atoms are shown (in green).

To rationalize the experimental observations, the electronic absorption spectrum calculations were carried out via time-dependent DFT. According to our previous studies [36,37], the cationic complex may adopt either a meridional (*a*) or a facial (*b*) isomeric form (Figure 3), wherein (*a*) the *N*-ethyl-*N*-(2-aminoethyl)salicylaldiminate (salEen) ligand moieties are not bent and adopt a meridional coordination mode, or (*b*) they are bent and form a coordination core with an O−Fe−O axis and two N−Fe−N(H) axes, i.e., the two N- or the two N(H)-donor atoms are *cis* to one another (Figure 3). Note that a facial *trans*-counterpart with the O−Fe−O, N−Fe−N, and N(H)−Fe−N(H) axes is the most energetically unfavorable isomeric form and, therefore, its formation is highly unlikely. If the salEen ligand is not functionalized by an extended periphery, it coordinates without bending to form the most stable *mer* isomer. Otherwise, a *fac* isomer may provide effective supramolecular interactions, and the overall energy balance in the crystalline lattice or the solution medium favors the formation of a less stable isomeric form. Since it is not obvious which of the two isomeric forms (Figure 3) the complex adopts in solution, the electronic absorption spectra were calculated for both of them.

The results of these calculations are presented in Figures S13 and S14, and Tables S1 and S2. To facilitate the analysis, the concept of natural transition orbitals (NTOs) was employed. The calculated electronic absorption spectra (Figures S13 and S14) were similar for both isomers, and were in good agreement with the experimental ones. Both HS (*S* = 5/2) and LS (*S* = 1/2) states were considered in the calculations, but no significant changes were detected in the corresponding spectra (Figures S13 and S14). Thus, based on the UV–Vis spectroscopic data alone, we cannot say which of the isomers is found in solution, nor is any clue given as to which of the two spin states the Fe(III) ions adopt at room temperature.

The calculated absorption wavelengths for the most intense electronic transitions can be grouped into three subsets, marked as A, B, and C in Figures S1 and S2. The electronic transitions of the first subset (A) are responsible for the experimentally observed low-energy absorption band. Several NTO pairs are required in order to describe the corresponding excited states (Tables S1 and S2). The occupied NTOs are composed of *π* carbazole-based orbitals, while the unoccupied NTOs consist of *π* benzoyloxy- and/or salicylidene-based orbitals, as well as 3d(Fe) orbitals. Thus, the electronic transitions of the first subset are composed of π–π* carbazole-to-benzoyloxy and carbazole-to-salicylidene charge transfers (ILCT), and *π–d* carbazole-to-metal charge transfers (LMCT).

The electronic transitions of the second subset (B) are composed of various π–π* (ILCT), *π–d* (LMCT), and *d–d* (MMCT) charge transfers, as well as of π–π* excitations centered at one carbazole moiety (IL transitions). The electronic transitions of the third subset (C) form the intense high-energy absorption band. The main responsible excitations for this absorption band are π–π* IL transitions. The calculated excitation energies for these electronic transitions are in good agreement with the experimentally observed absorption regions.

Finally, the results of our calculations suggest that carbazole moieties—or rather, π_carb_–π* intraligand (ILCT) and *π_carb_*–d_Fe_ ligand-to-metal (LMCT) charge transfers—are responsible for a double-band emission observed for our compounds.

### 3.6. X-ray Powder Diffraction Analysis

The calculated crystallographic parameters of compounds **1**–**3**, in accordance with the phase identification based on the results of calculations performed using the EVA and TOPAS software packages, are presented in Table 1. Experimental diffraction patterns are presented in Figures S15–S17.

For all compounds, the crystalline lattice has a triclinic symmetry, space group P1. The unit cell contains two complex cations, which adopt facial isomeric form. The packing density increases with an increase in the counterion mass. This is due to the fact that anions are embedded in a cell with positively charged molecular ions.

### 3.7. Magnetic Properties

The magnetic properties of compounds **1**–**3** were studied via SQUID magnetometry and EPR spectroscopy. While SQUID is an integral magnetic method, EPR is a locally sensitive technique and a powerful instrument for the detection and analysis of the temperature dependences of the EPR intensity of the individual contributions of the HS and LS Fe(III) ions in different environments.

#### 3.7.1. EPR Spectroscopy

**Figure 4:**
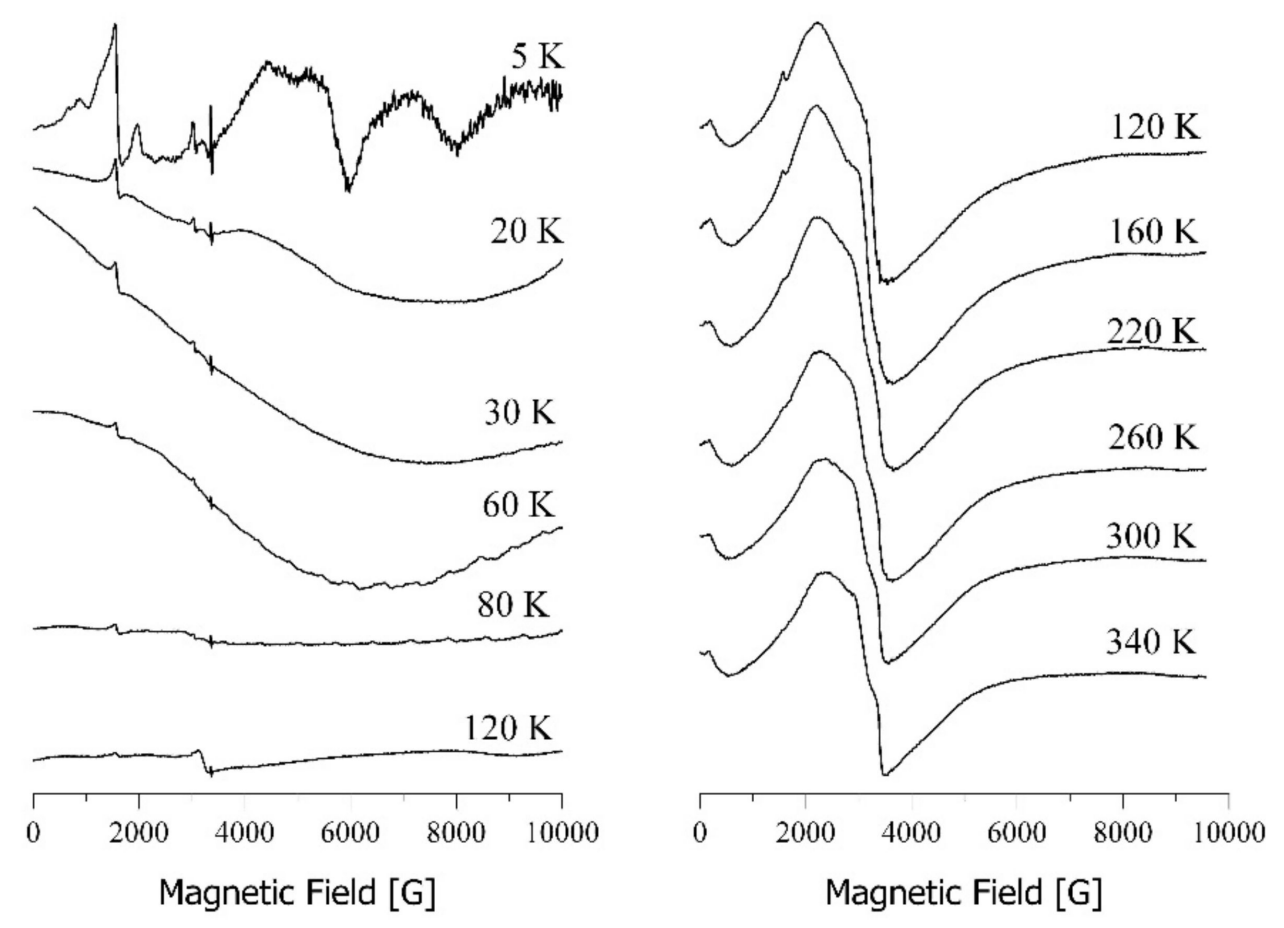
The temperature dependence of the EPR X-band spectra of **1** from 5 K to 340 K.

**Figure 5:**
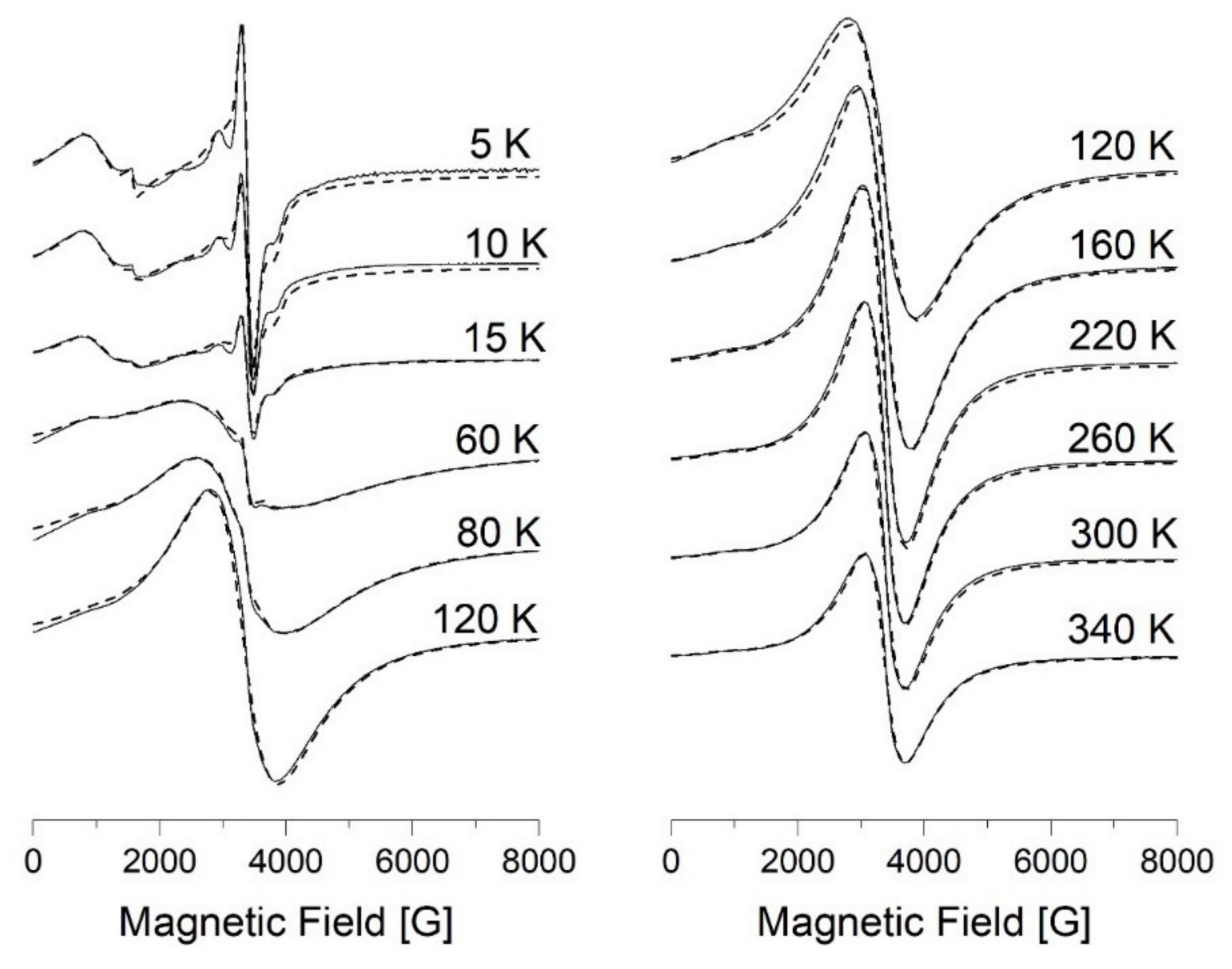
The temperature dependence of the EPR X-band spectra of **2** from 5 K to 340 K. The dashed lines show the theoretical EPR spectra.

**Figure 6:**
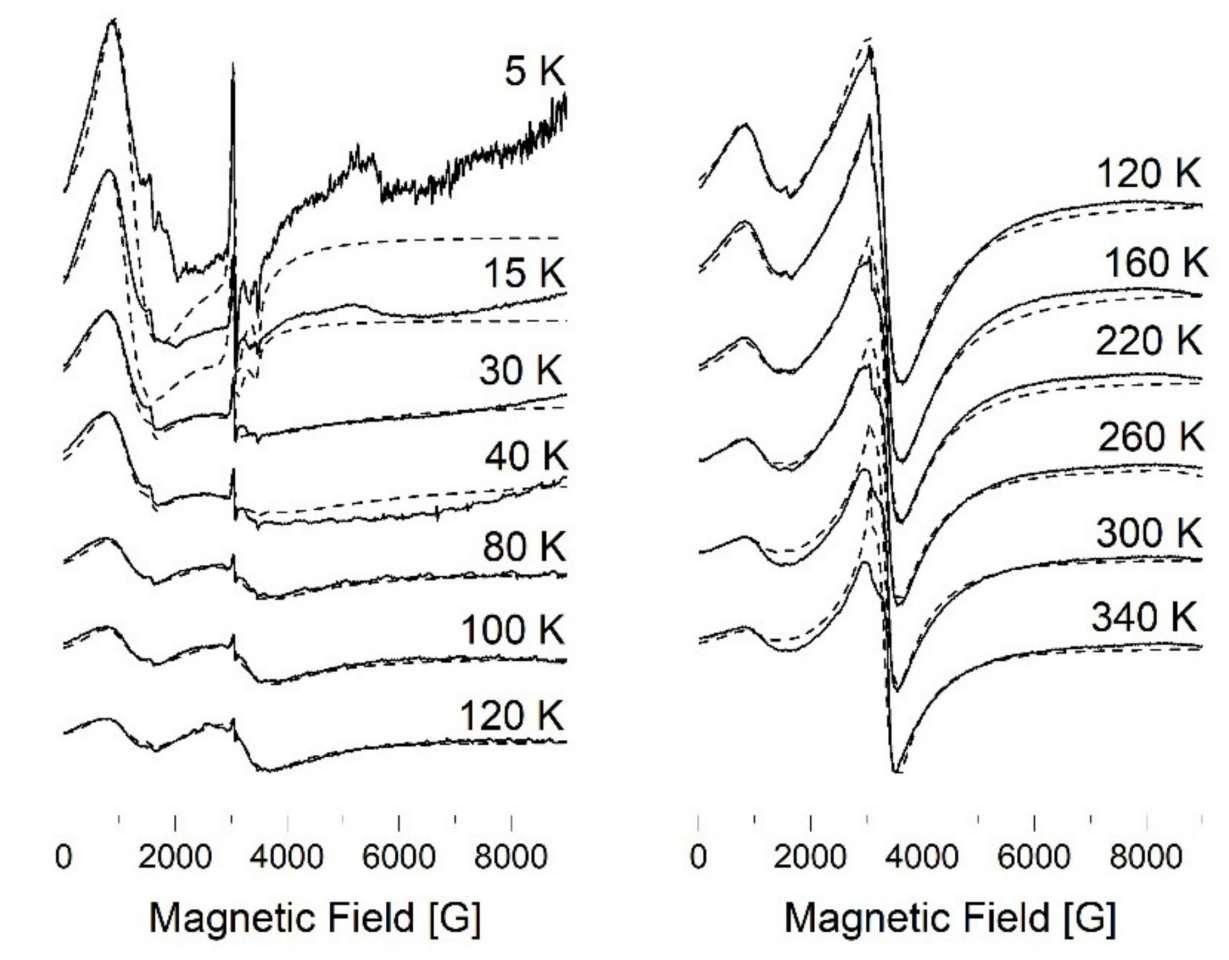
The temperature dependence of the EPR X-band spectra of **3** from 5 K to 340 K. The dashed lines show the theoretical EPR spectra.

EPR spectroscopy is a powerful technique used to obtain qualitative information about the presence of HS and LS fractions, their evolution with temperature, and to confirm the presence of antiferromagnetic (AFM) interactions. EPR measurements were carried out in the temperature range of 5–340 K. The most illustrative X-band EPR spectra (hν = 0.3 cm^–1^) are shown in Figure 4, Figure 5 and Figure 6 for compounds **1**, **2**, and **3**, respectively.

The most difficult shape of the spectra is observed for **1** (Figure 4). At room temperature, the intense broad signal has a complex shape—probably due to the superposition of different low-intensity unseparated signals. Since the intensity of the main resonance is wiped out at temperatures below 100 K, several weak and narrow resonance lines can be discerned—a low-intensity HS signal with *g*_eff_ ~ 4.2, and an LS signal. Numerous noise-like low-intensity signals are observed at 5 K; they broaden as the temperature increases, and a very broad line is observed in the temperature range from 20 to 80 K. Such signals are observed in the empty open EPR quartz tube, and the cause of these signals is most likely the frozen atmospheric oxygen [38]. For other compounds, these signals are of very low intensity, and are not visible against the background of intense sample signals. A very broad signal, probably of the same nature, is also observed in the spectra of **3** (from 20 to 80 K) (Figure 6).

At 340 K, the spectra of all three compounds are similar, and consist of an intense broad line with *g*_eff_ ~ 2 and Δ*H* = 1128, 640, and 456 G for **1**, **2**, and **3**, respectively. The broad resonance line is typical for HS (*S* = 5/2) ions with a weakly distorted octahedral environment (|*D*| < *hν* = 0.3 cm^–1^, *E* = 0) (III-type of HS centers). With decreasing temperature, the line widens and shifts towards weaker fields.

As the temperature increases, signals with *g*_eff_ = 6, *g*_eff_ ~ 4.2, and narrow anisotropic LS (*S* = 1/2) signals with *g*_x_ = 2.01, *g*_y_ = 2.01, and *g*_z_ = 1.75 become distinguishable, and grow in intensity. The signal with *g*_eff_ = 6 belongs to HS Fe(III) ions in a strong axial crystal field (|*D*| >> *hν* = 0.3 cm^–1^, *E* = 0) (I-type of HS centers), whereas the signal with *g*_eff_ = 4.2 belongs to HS Fe(III) ions in a strong (*D* > *hν* = 0.3 cm^–1^) low-symmetry (*E*/*D* = 1/3) crystal field (II-type of HS centers) acting on the iron site from the middle of the three Kramers doublets [39,40].

**Figure 7:**
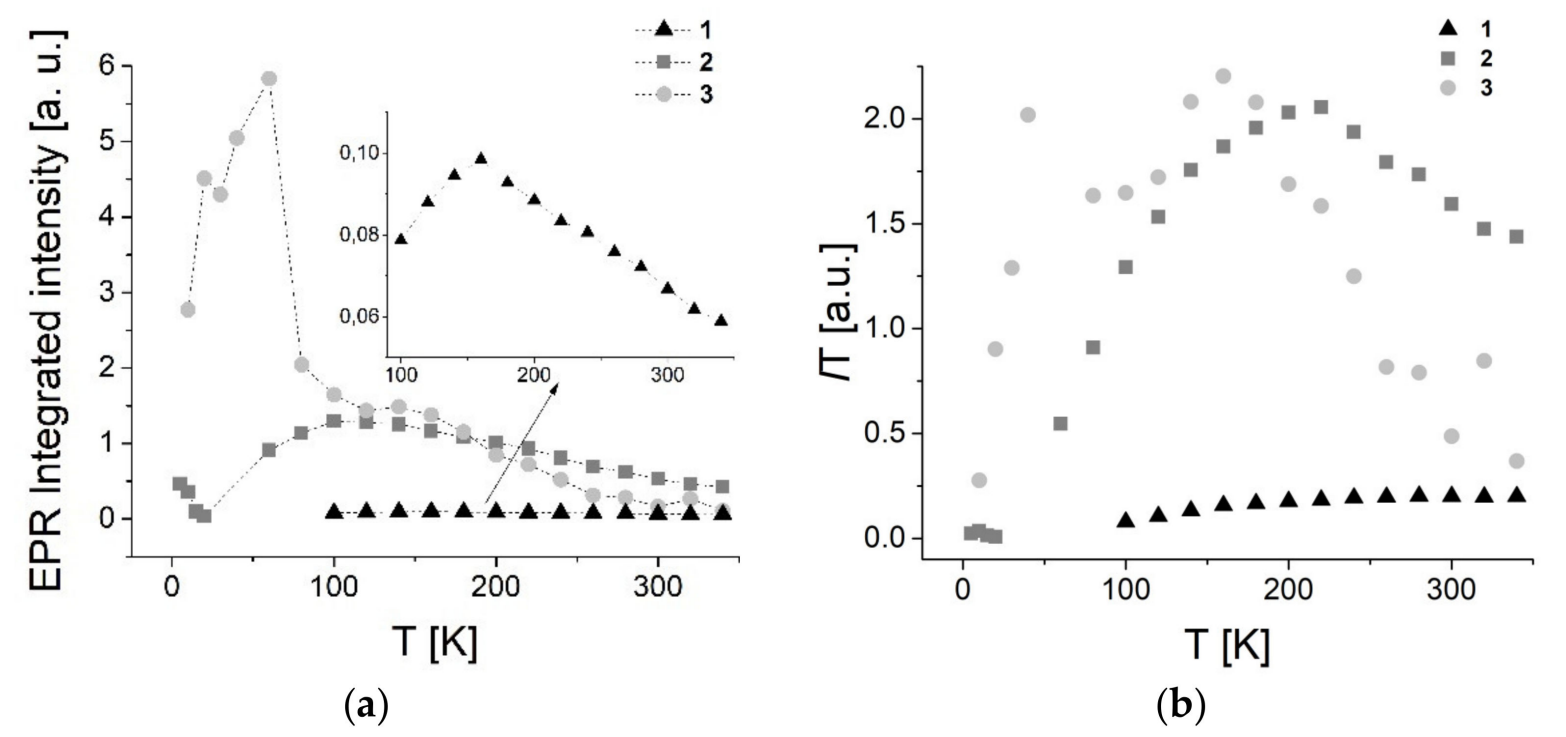
The temperature dependencies of the EPR lines of the integrated intensity *I* of the whole spectra for all three compounds (**a**), and the *IT* product (**b**).

The temperature dependencies of the integrated intensity *I* of the whole spectra—which is obtained via numerical double-integration of the EPR spectra, and is proportional to the static paramagnetic susceptibility—are shown in Figure 7 for all three compounds. One can see that the temperature dependence of *I* passes through a broad maximum at *T*_max_ ≈ 160, 120, and 130 K for **1**, **2**, and **3**, respectively. The abnormal increase and decrease in the integral intensity for **3** in the temperature range from 20 to 80 K is probably due to the superposition of a very broad signal from the frozen atmospheric oxygen.

**Figure 8:**
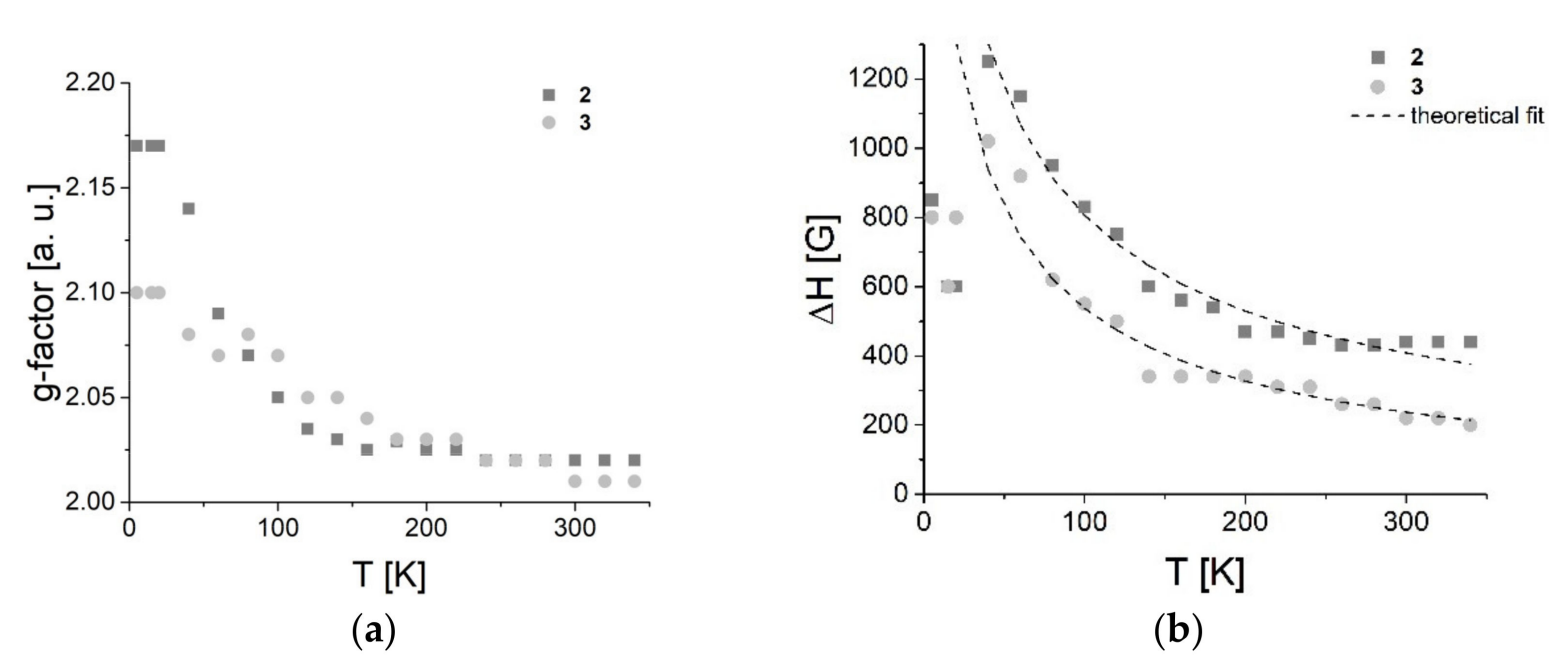
The temperature dependences of the resonance magnetic field (**a**) and line width (**b**) of III-type HS Fe(III) centers in **2** and **3**. The dashed lines show the theoretical fit.

To understand the unusual behavior of the temperature dependence of the EPR spectra’s integrated intensity, we decomposed the spectra and studied the properties of the Fe(III) centers separately. The procedure of decomposition was carried out using the EasySpin MATLAB program [41,42] with the following magnetic parameters: I-type HS centers—*D* ≥ 1.49 cm^–1^, *E* = 0 cm^–1^, *g* = 2, Δ*H* = 720 G; II-type HS centers—*D* = 0.42 cm^–1^, *E* = 0.1 cm^–1^, *g* = 2, Δ*H* = 70–100 G; III-type HS centers—*D* = 0.025 cm^–1^, *E* = 0.1 cm^–1^, variations of *g* and Δ*H* are shown in Figure 8 for **2** and **3**; LS centers—*g*_x_ = 2.01, *g*_y_ = 2.01, *g*_z_ = 1.75 (for **2**, a second LS signal is also observed with *g*_x_ = 1.96, *g*_y_ = 1.96, and *g*_z_ = 2).

**Figure 9:**
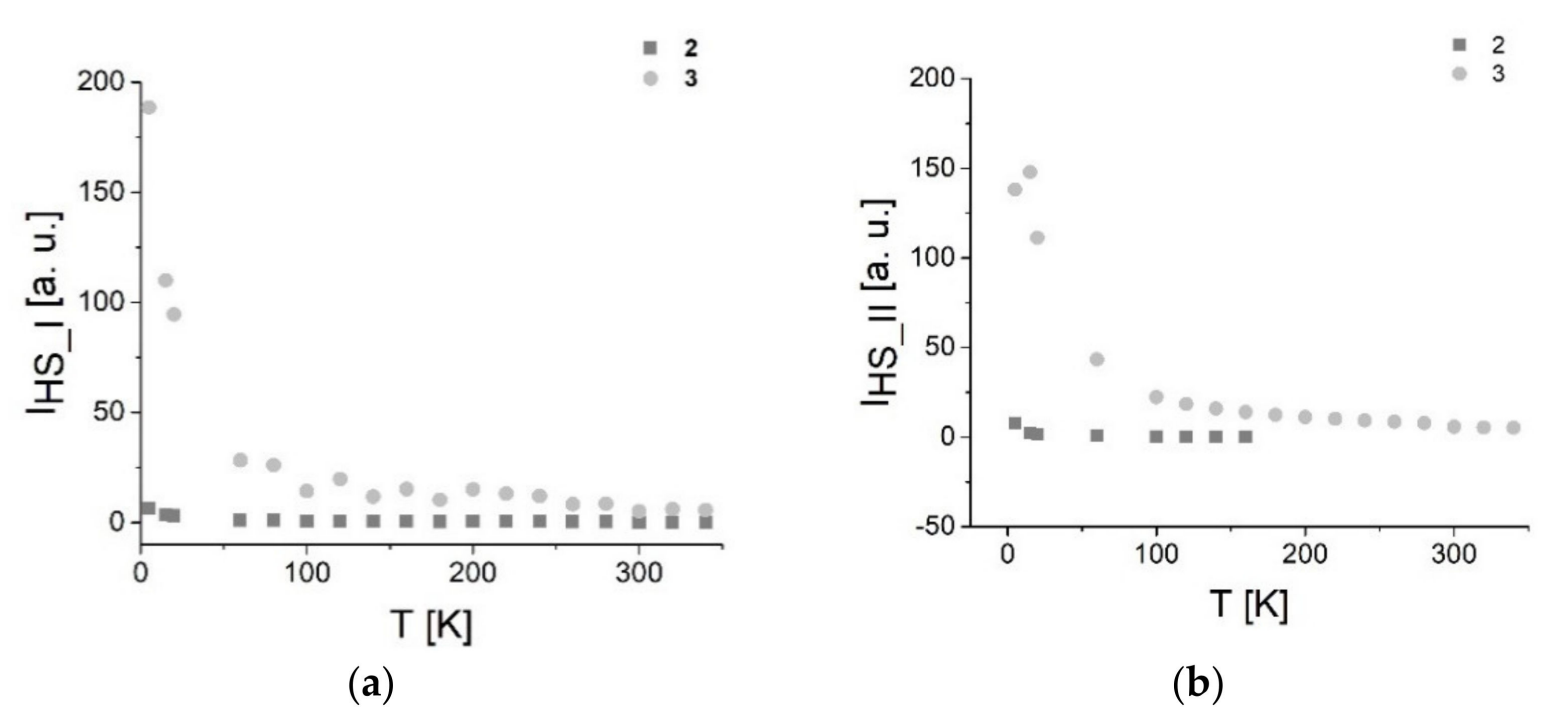
The temperature dependences of the integrated intensity of I-type (**a**) and II-type (**b**) HS Fe(III) centers in **2** and **3**.

**Figure 10:**
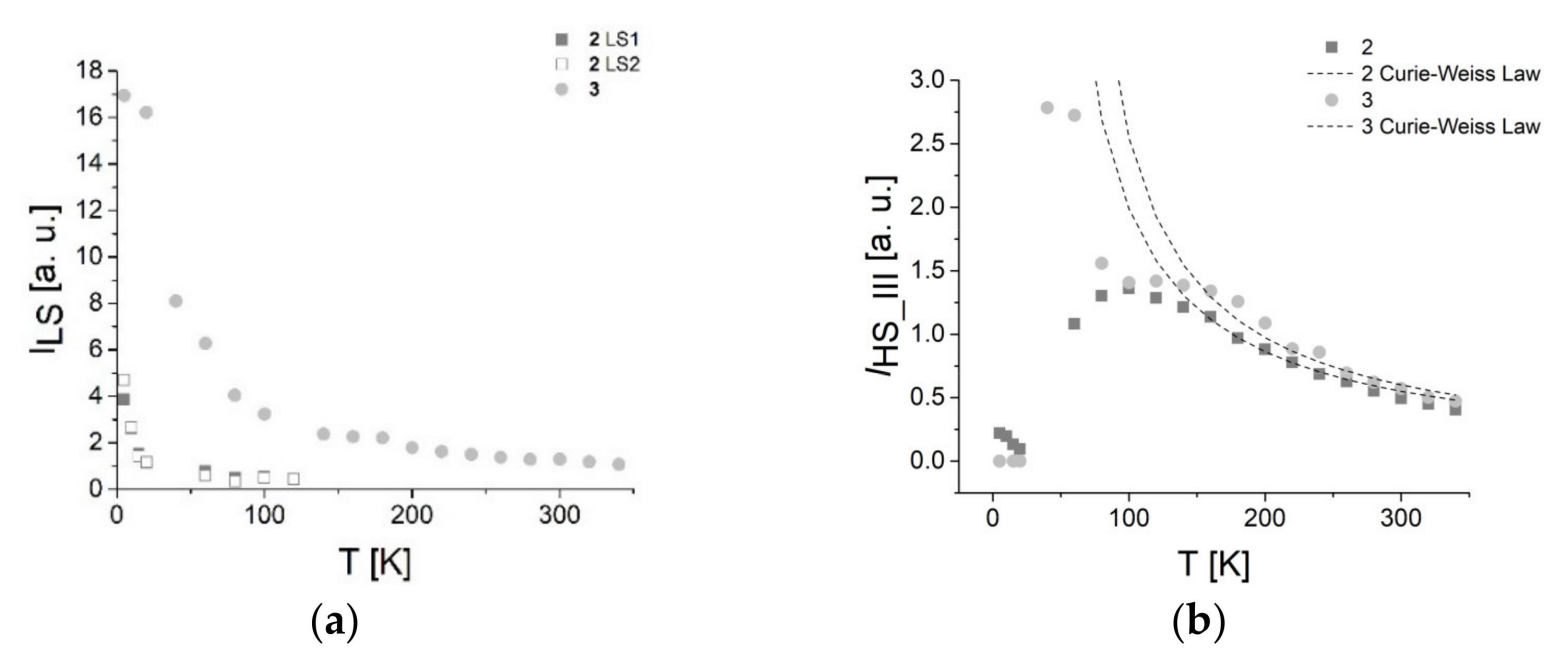
The temperature dependences of the integrated intensity of LS (**a**) and III-type HS (**b**) Fe(III) centers in **2** and **3**. The dashed lines are calculated within the Bonner–Fisher model for antiferromagnetically coupled chains.

The temperature dependences of the integrated intensities for I-type and II-type HS centers (Figure 9) and LS centers (Figure 10) demonstrate sharp maxima at 4 K, and follow the Curie–Weiss law in the whole temperature range. We can assume that the number of these centers is small and, therefore, that these centers are isolated individual paramagnetic ions. The EPR signal from LS complexes is not observed, due to short spin-lattice relaxation times [43].

The integrated intensity of the III-type HS centers (measured as the *I*Δ*H*^2^ product) has a maximum at *T* ≈ 100 and 120 K for **2** and **3** (Figure 10). The temperature dependence of this line of integrated intensity in the temperature range from 160 K to 340 K can be described by the Curie–Weiss law with the value of the Weiss constant *θ* = −23 and −38 K for **2** and **3**. Some features of the signal in the range of 160–300 K (temperature-independent position in the field, correspondence of the temperature dependence of the integral signal intensity to the Curie–Weiss law) suggest that the sample is paramagnetic within this temperature range. The negative sign of the Weiss constant indicates the antiferromagnetic character of the interaction between Fe(III) ions with a weakly distorted octahedral environment at temperatures below 100 K.

At temperatures below 100 K, the observed magnetic resonance signal has features characteristic of the antiferromagnetic resonance line (a large line width, an increase in the line width, and a line shift towards weak fields with decreasing temperature). The sharp increase in the line width with a decrease in temperature below 100 K may be due to an increase in fluctuations of the magnetic moment as the temperature of magnetic ordering (*T*_N_) is approached [43]. In this case, the temperature dependence of the line width is described by Equation (1) [44]:

(1) $$\Delta H=\Delta H_{0}+A{\left[T-T_{c}\right]}^{-0.7}$$

with *T*_N_ = −20 K, Δ*H*_0_ = 4.7 G, *A* = 22,929 G K^0.7^ for **2,** and *T*_N_ = −15 K, Δ*H*_0_ = −55 G, *A* = 16,400 G K^0.7^ for **3** (Figure 8).

#### 3.7.2. SQUID Magnetometry

**Figure 11:**
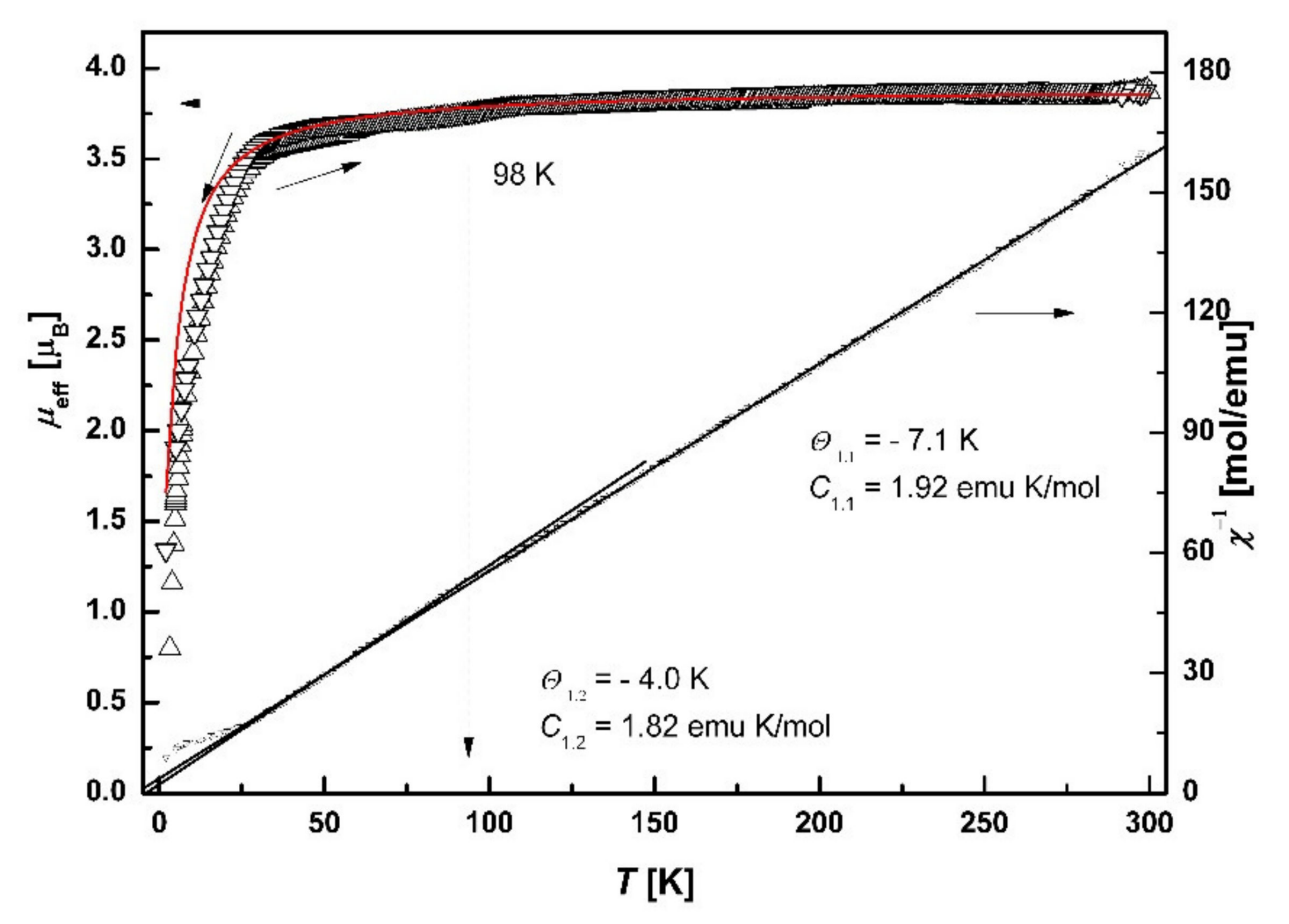
The temperature dependences of the effective magnetic moment, *μ*_eff_, and the inverse magnetic susceptibility, *χ*^–1^, for **1**. Solid lines correspond to the Curie–Weiss fit with *Θ*_1.1_ = −7.1 K (110 < *T* < 300 K) and *Θ*_1.2_ = −4.0 K (30 < *T* < 90 K). The solid red line represents the best fit within the Bonner–Fisher model for LS and HS chains (*J*_1_^LS^ = −12.0 K, *J*_1_^HS^ = −0.93 K), including PM contribution.

**Figure 12:**
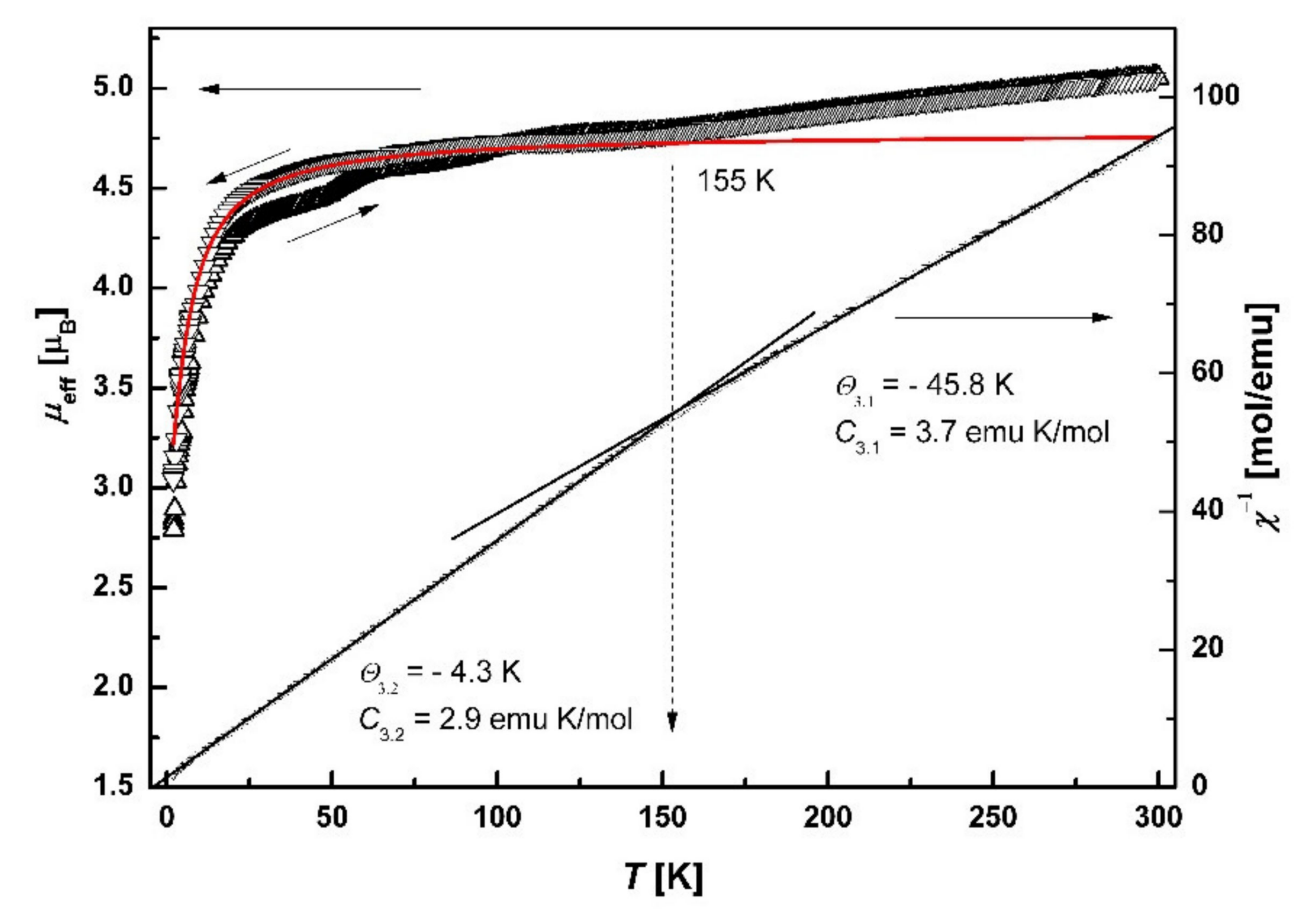
The temperature dependences of the effective magnetic moment, *μ*_eff_, and the inverse magnetic susceptibility, *χ* ^–1^, for **3**. Solid lines correspond to the Curie–Weiss fit with *Θ*_3.1_ = −45.8 K (180 < *T* < 300 K) and *Θ*_3.2_ = −4.3 K (7 < *T* < 140 K). The solid red line represents the best fit within the Bonner–Fisher model for HS chains (*J*_3_^HS^ = −1.0 K), including PM contribution.

**Figure 13:**
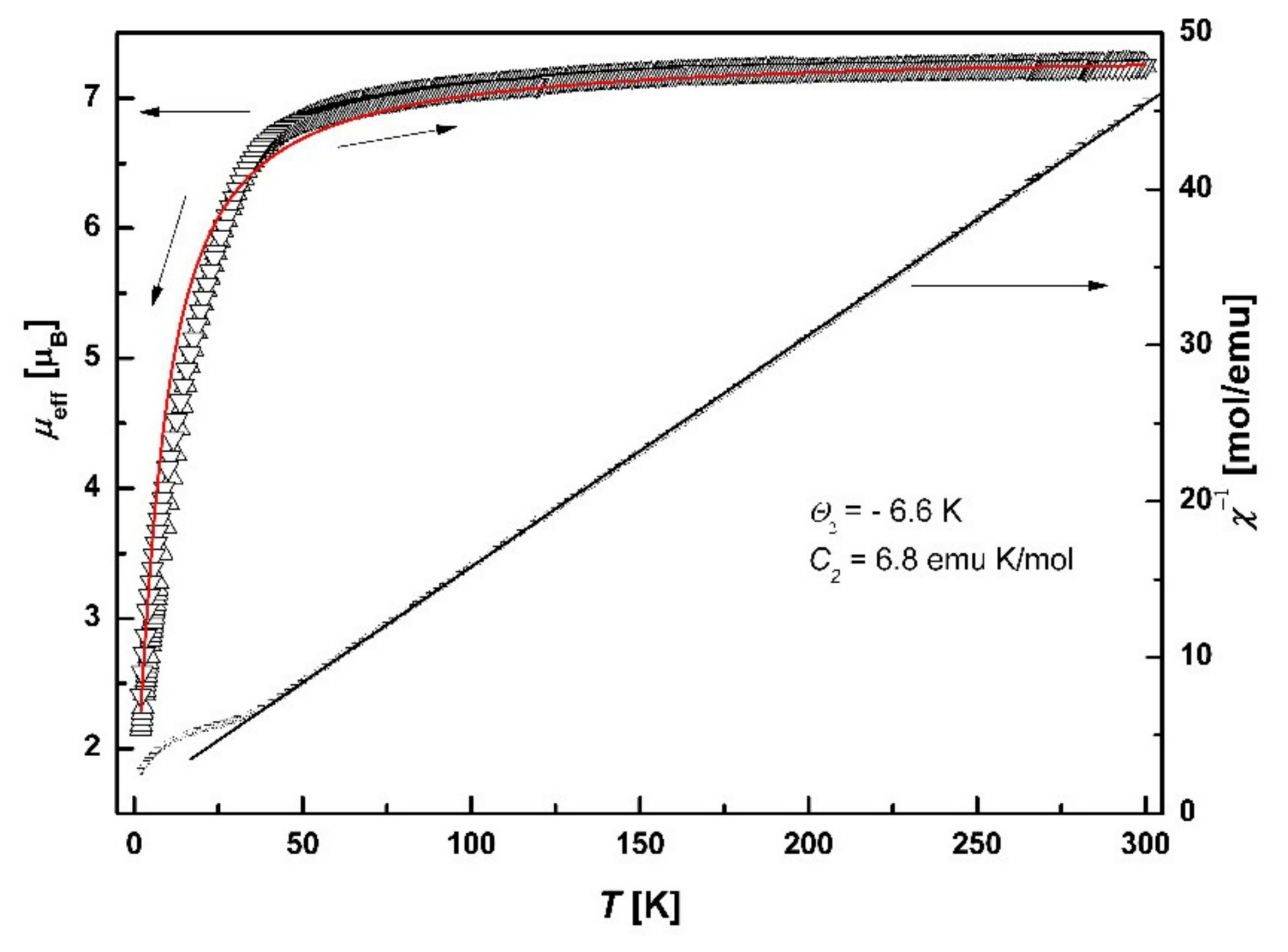
The temperature dependences of the effective magnetic moment, *μ*_eff_, and the inverse magnetic susceptibility, *χ*^–1^, for **2**. The solid line corresponds to the Curie–Weiss fit with *Θ*_2_ = −6.6 K. The solid red line represents the best fit within the Bonner–Fisher model for HS chains (*J*_2_^HS^ = −1.8 K), including PM contribution.

The temperature evolution of the static magnetic susceptibility, *χ*, in the form of the effective magnetic moment, *μ*_eff_, for **1**, **3**, and **2** under heating (Δ) and cooling (∇) regimes are shown in Figure 11, Figure 12 and Figure 13, respectively.

In all cases, the thermocycling reveals the irreversibility of the magnetic moment (small hysteresis). Intricate hysteresis of spin susceptibility is typical for mononuclear SCO iron(III) complexes [45]. Note that further analysis concerns the magnetic data obtained only under the cooling (∇) regime. The magnetic susceptibility data for **1** demonstrate a gradual decrease in *μ*_eff_(*T*) (Figure 11), from 3.86 *μ*_B_ at 300 K to 1.34 *μ*_B_ at 2.0 K. The magnetic response is expected to result only from the contribution of Fe(III) ions, which can be in either LS (*S* = 1/2) or HS (*S* = 5/2) states. Since the experimental value of *μ*_eff_ is much less than the theoretical value for HS Fe(III) ions, but much higher than that for LS Fe(III) ions, a mixed spin state (HS + LS) can be assumed. Using Equation (2) [46]:

(2) $$\mu_{eff}^{2}=x\mu_{eff}^{2}(LS)+(1-x)\mu_{eff}^{2}(HS),$$

where *μ*_eff_(LS) = 1.73 *μ*_B_ is the theoretical value for LS Fe(III) ions, and *μ*_eff_(HS) = 5.91 *μ*_B_ − for HS Fe(III) ions (*g* = 2.0), while *x* is the fraction of LS species, we estimated the LS and HS fractions, which were approximately 62% and 38%, respectively, at room temperature. The *μ*_eff_ value remains nearly temperature-independent in the range of 110–300 K. Between 110 and 90 K, the *μ*_eff_ value decreases to attain a minimum of 3.74 *μ*_B_ at 90 K (note that under the heating (Δ) regime a two-step increase is observed), corresponding to 66% of LS Fe(III) ions and 34% of HS Fe(III) ions. Thus, the slight increase in this range is most likely due to the occurrence of partial SCO. Upon further cooling, a drastic decrease in *μ*_eff_(*T*) is observed at *T* < 30 K. Such behavior is characteristic of AFM interactions and/or zero-field splitting (ZFS). For clarification, the temperature dependence of the inverse magnetic susceptibility, *χ*^–1^(*T*), was analyzed (Figure 11) under the cooling (∇) regime. It was found that the *χ*^–1^(*T*) data are well described by two Curie–Weiss laws with Weiss constants *Θ*_1.1_ = −7.1 K, *C*_1.1_ = 1.92 emu K mol^–1^ (110 < *T* < 300 K) and *Θ*_1.2_ = −4.0 K, *C*_1.2_ = 1.82 emu K mol^–1^ (30 < *T* < 90 K). Such behavior of *χ*^–1^(*T*) indicates partial SCO with *T*_1/2(1)_ = 98 K. The negative sign of *Θ* indicates the presence of AFM interactions between Fe(III) ions. The ZFS has a minor impact on the decrease in *μ*_eff_(*T*), since the contribution of the ZFS parameter *D* to the Weiss constant is small [47].

The substitution of NO_3_^−^ by PF_6_^−^ does not lead to a drastic change in the *μ*_eff_(*T*) behavior, but results in a considerable increase in *μ*_eff_(*T*) (Figure 12). Compound **3** demonstrates a gradual decrease in *μ*_eff_(*T*), from 5.05 *μ*_B_ at 300 K to 2.8 *μ*_B_ at 2.0 K. The estimation of the LS and HS fractions according to Equation (2) gives 28% and 72%, respectively, at room temperature. The *μ*_eff_ value slightly decreases in the range of 180–300 K. At *T* ≈ 160 K, the inflection point is observed, and the *μ*_eff_ value attains a minimum of 4.75 *μ*_B_ at 140 K (note that under the heating (Δ) regime a three-step increase is observed), corresponding to 39% of LS Fe(III) ions and 61% of HS Fe(III) ions. Upon further cooling, a drastic decrease in *μ*_eff_(*T*) is observed at *T* < 7.0 K. It was found that the *χ*^–1^(*T*) data (Figure 12) are also well described by two Curie–Weiss laws with Weiss constants *Θ*_3.1_ = −45.8 K, *C*_3.1_ = 3.7 emu K mol^–1^ (180 < *T* < 300 K) and *Θ*_3.2_ = −4.3 K, *C*_3.2_ = 2.9 emu K mol^–1^ (7 < *T* < 140 K). Thus, we can conclude that a partial SCO probably takes place at *T*_1/2(3)_ = 155 K, and a decrease in *μ*_eff_ is caused by AFM interactions between Fe(III) ions.

Compound **2** is characterized by the largest value of *μ*_eff_ = 7.25 *μ*_B_ at 300 K (Figure 13), which is higher than the theoretical value for HS Fe(III) ions. This may be due to *g* > 2.0. Upon cooling, *μ*_eff_ demonstrates a decrease to the value of 2.41 *μ*_B_ at 2.0 K. The *χ* ^–1^(*T*) plot follows the Curie–Weiss law in a wide temperature range with *Θ*_2_ = −6.6 K, *C*_2_ = 6.8 emu K mol^–1^ (Figure 13). Since no deviation from the Curie–Weiss law is observed, we can conclude that there is no spin transition in **2**. Note also that the SQUID data demonstrate the presence of AFM interactions between Fe(III) ions at low temperatures.

**Figure 14:**
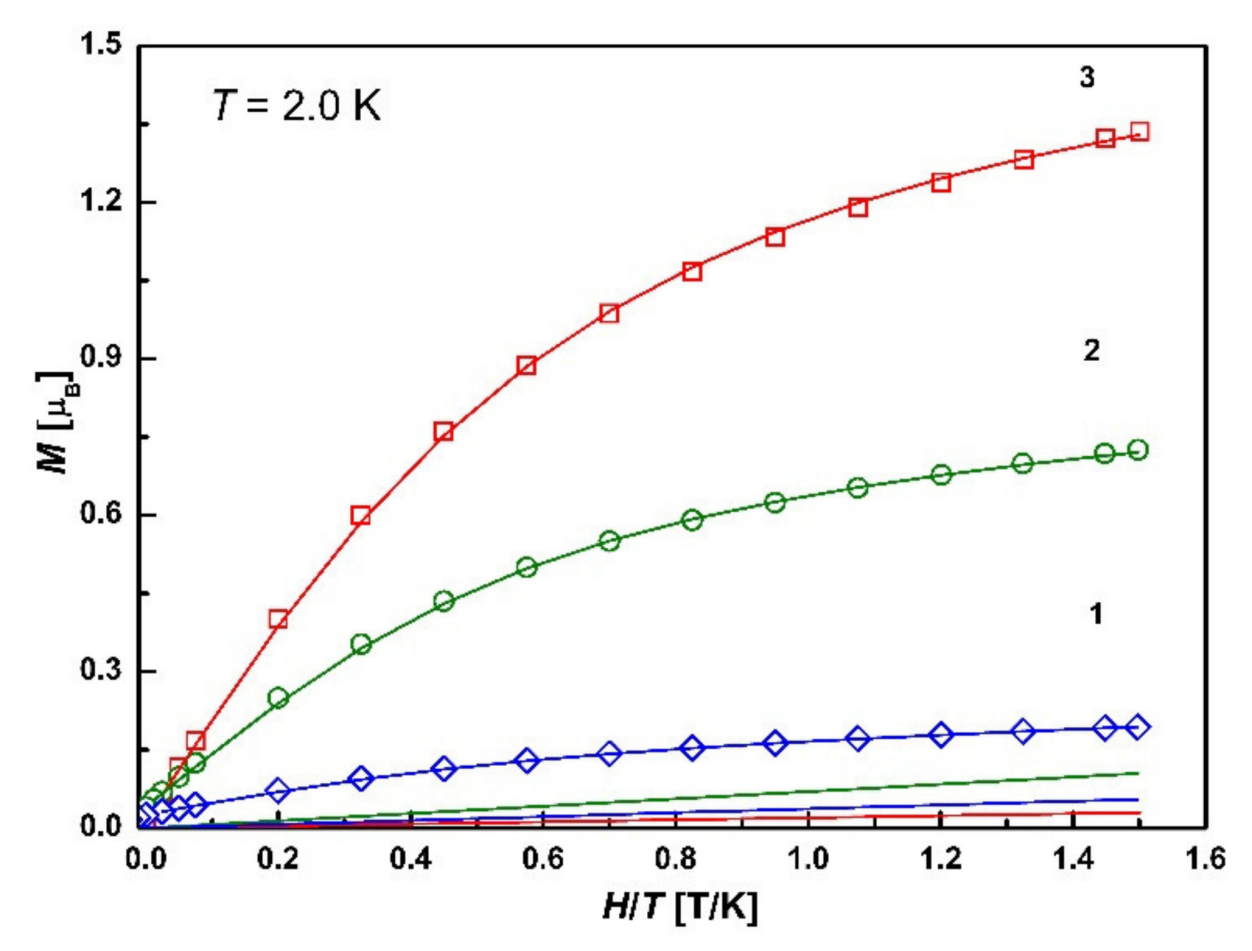
The field dependences of the magnetization, *M*, measured at *T* = 2.0 K for **1** (◊), **2** (ο), and **3** (□). Solid lines are the best fit curves corresponding to a superposition of linear function (straight lines) and weighted Brillouin functions for *S* = 5/2 and *S* = 1/2 (see main text for details).

To determine the ground spin state for **1** (◊), **2** (ο), and **3** (?), the field dependences of the magnetization, *M*(*H*), were measured at 2.0 K (Figure 14).

All of the curves exhibit a paramagnetic-like behavior, but the saturation value at the highest fields was not reached, and the trend of its linear growth was distinguished. The absence of a flat saturation segment *M*(*H*) = *M*_s_ at the highest fields could be attributed to a process of canting of the antiferromagnetically coupled spins. The magnetization curves cannot be approximated by the simple Brillouin functions for *S* = 5/2 or *S* = 1/2; they were perfectly fitted by their combination with the corresponding weight factors, and by taking into account a linear function accounting for AFM interactions. The corresponding fitting curves (solid lines) are presented in Figure 14; AFM contributions are depicted separately (straight lines). Fitting parameters are presented in Table 2.

The Brillouin functions indicate that some of the local moments of Fe(III) ions remain non-interacting in the paramagnetic (PM) state. The ground spin states for **1**–**3** are different. For **2**, the PM fraction of HS Fe(III) ions is 10%, while for **1** and **3**, the fractions are 2.5% HS and 21% HS, 39% LS, respectively. Note that the 39% LS fraction for **3** coincides with the value determined from *μ*_eff_ at 140 K after a partial spin transition. This suggests that LS Fe(III) ions are not involved in AFM interactions. For **1**, the LS fraction decreases from 66% at 90 K to 0% at 2.0 K. Thus, all LS Fe(III) ions are in the AFM state.

The structure of compounds **1**–**3** and their mixed spin states suggest two mechanisms of AFM interactions between the neighboring Fe(III) ions: spin chains formed by LS and HS Fe(III) ions with a weakly distorted octahedral environment, and/or dimeric ensembles of HS–HS, LS–LS formed by LS and HS Fe(III) ions with a strongly distorted octahedral environment. In general, determination of such a variety of exchange interactions seems to be a nontrivial problem. Therefore, attempts were made to describe *χ*(*T*) within the Bonner–Fisher and Bleaney–Bowers models. In the first case, the equation describing the temperature dependence *χ*(*T*) for linear chains of classical spins with *S* = 5/2 is given as [48]:

(3) $$\chi =\frac{Ng^{2}\mu_{B}^{2}S(S+1)}{3k_{B}T}\left(\frac{1+u}{1-u}\right),\;u=\coth\left(\frac{2JS(S+1)}{k_{B}T}\right)-\frac{k_{B}T}{2JS(S+1)};$$

with *S* = 1/2 – as [49]:

(4) $$\chi =\frac{Ng^{2}\mu_{B}^{2}}{k_{B}T}\frac{0.25+0.074975x+0.075235x^{2}}{1+0.9931x+0.172135x^{2}+0.757825x^{3}},\;x=\frac{\left|J\right|}{k_{B}T}.$$

In the second case, the equation describing *χ*(*T*) of the dimeric phase is [50]:

(5) $$\chi =\frac{Ng^{2}\mu_{B}^{2}}{3\;k_{B}T}\frac{\sum_{S}S(S+1)(2S+1)e^{-E(J,S)/k_{B}T}}{\sum_{S}(2S+1)e^{-E(J,S)/k_{B}T}},$$

where $E(J,S)=-J[S(S+1)-S_{a}(S_{a}+1)-S_{b}(S_{b}+1)]$, $S=S_{a}+S_{b},S_{a}+S_{b}-1,\ldots ,\left|S_{a}-S_{b}\right|$ is the total spin value.

In the case of *S*_a_ = 5/2 and *S*_b_ = 5/2, the Bleaney–Bowers equation can be reduced to the expression:

(6) $$\chi =\alpha \frac{Ng^{2}\mu_{B}^{2}}{3k_{B}T}\frac{330e^{12.5J/k_{B}T}+180e^{2.5J/k_{B}T}+84e^{-5.5J/k_{B}T}+30e^{-11.5J/k_{B}T}+6e^{-15.5J/k_{B}T}}{11e^{12.5J/k_{B}T}+9e^{2.5J/k_{B}T}+7e^{-5.5J/k_{B}T}+5e^{-11.5J/k_{B}T}+3e^{-15.5J/k_{B}T}+e^{-17.5J/k_{B}T}}.$$

In the case of *S*_a_ = 1/2 and *S*_b_ = 1/2, we get:

(7) $$\chi =\alpha \frac{2Ng^{2}\mu_{B}^{2}}{k_{B}T}\frac{1}{\left(3+\exp(-2J/k_{B}T)\right)},$$

where *α* is the factor taking into account a deviation of the Fe(III) *g*-factor.

Numerical simulation showed that the best fitting (solid red lines in Figure 11, Figure 12 and Figure 13) is obtained within the Bonner–Fisher model, taking into account the PM contribution, *χ*_pm_, of Fe(III) ions, which are not involved in AFM interactions, estimated from *M*(*H*) data:

(8) $$\chi =\chi_{1/2chain}+\chi_{5/2chain}+\chi_{pm},$$

where for **1** there are spin chains with *S* = 1/2 and 5/2, while for **2** and **3** there are only chains with *S* = 5/2. The corresponding exchange constants *J*^LS^ and *J*^HS^ were estimated: **1**—*J*_1_^LS^ = −12.0 K, *J*_1_^HS^ = −0.93 K; **2**—*J*_2_^HS^ = −1.8 K; **3**—*J*_3_^HS^ = −1.0 K.

Thus, it can be concluded that, at room temperature, the majority of Fe(III) ions in **2** are in the HS state, and the SCO transition is completely suppressed, while **1** and **3** are characterized by a mixed spin state (HS + LS), and show a partial spin transition at *T*_1/2(1)_ = 98 K and *T*_1/2(3)_ = 155 K, respectively. All three compounds exhibit AFM interactions between the neighboring Fe(III) ions, which probably form spin chains (S = 1/2; 5/2) in layers. It was shown that not all Fe(III) ions are involved in AFM interactions in the ground spin state at 2.0 K—rather, some of them (HS and LS) are in the PM state.

## 4. Conclusions

A series of azomethine complexes of the composition [Fe(L)_2_]X, where L = 3,6-bis[(3′,6′-di-*tert*-butyl-9-carbazol)-9-carbazol]benzoyloxy-4-salicylidene-*N*′-ethyl-*N*-ethylenediamine, X = NO_3_^−^, Cl^−^, PF_6_^−^ (**1**–**3**), were synthesized and characterized. For all of the compounds, the crystalline lattice has a triclinic symmetry, space group P1, while the unit cell contains two complex cations, which adopt facial isomeric form.

The study of thermal properties via TG/DTG and DSC techniques allows us to conclude that the nature of phase transitions is determined by the nature of the counterion. Compound **1** is characterized by “solid–solid” type phase transitions reproducible under heating and cooling cycles. When the counterion is replaced by metathesis by the PF_6_^−^ ion (**3**), the system undergoes a series of solid-state transitions. Compound **2**, synthesized from the iron(III) chloride salt, exhibits a different behavior. No crystalline transitions were detected by DSC measurements; only the amorphous state of the sample with a glass transition temperature of 143 °C was found. Within the whole series of compounds, no melting or softening point was observed. It was found that all of the compounds have high thermal stability up to 250 °C.

The UV–Vis absorption and fluorescence properties were studied in dichloromethane at room temperature. To obtain insight into the spectroscopic characteristics, the electronic absorption spectrum calculations were carried out via time-dependent DFT. An intense absorption band centered in the UV region at ~240 nm was attributed to π–π* intraligand transitions. The next absorption band, centered at ~300 nm, was attributed to various *π*–π* intraligand, *π*–*d* ligand-metal, and *d–d* metal-to-metal charge transfers, as well as to *π*–π* intracarbazole transitions. Finally, the lowest energy absorption band centered at ~335 nm was attributed to *π*–π* carbazole-to-benzoyloxy and carbazole-to-salicylidene charge transfers (ILCT), and *π*–d carbazole-to-metal charge transfers (LMCT). Upon excitation at 350 nm, the complex exhibits an intense dual fluorescence with two emission bands centered at ~445 and ~485 nm. According to our calculations, carbazole moieties—or rather, *π*_carb_–π* intraligand and *π*_carb_–d_Fe_ ligand-to-metal charge-transfer excited states—are responsible for the observed dual emission pattern.

EPR spectroscopy and SQUID magnetometry were used to study the magnetic properties and the influence of the counterion on the structure and the spin transition. Magnetic measurements revealed that, at room temperature, compound **1** contains 62% low-spin (LS, *S* = 1/2) and 38% high-spin (HS, *S* = 5/2) Fe(III) ions, and undergoes a partial SCO (*S* = 5/2 → 1/2) at 98 K. Compound **2** is only in the HS state, and the SCO transition is completely suppressed. Compound **3** is also characterized by a mixed spin state (28% LS, 72% HS), and shows a partial spin transition at 155 K. All three compounds exhibit AFM interactions between the neighboring Fe(III) ions. In the framework of a spin chain model (*S* = 5/2 and 1/2), the exchange constants *J*^LS^ and *J*^HS^ were estimated: **1**—*J*_1_^LS^ = −12.0 K, *J*_1_^HS^ = −0.93 K; **2**—*J*_2_^HS^ = −1.8 K; **3**—*J*_3_^HS^ = −1.0 K. It was shown that not all Fe(III) ions are involved in AFM interactions in the ground spin state at 2.0 K—some of them (HS and LS) are in the paramagnetic state.

## Figures and Tables

**Table 1:** Crystallographic data for compounds **1**–**3**.

	[Fe(L)_2_]NO_3_	[Fe(L)_2_]Cl	[Fe(L)_2_]PF_6_
Symmetry	P1	P1	P1
a, Å	14.58	11.78	8.48
b, Å	11.93	15.49	6.12
c, Å	20.80	24.90	23.06
α, °	78.64	46.41	53.83
β, °	70.84	127.8	97.40
γ, °	24.86	136.6	105.1

**Table 2:** Fitting parameters for *M*(*H*) for **1**, **2**, and **3**.

№	*M*_s_, *μ*_B_	Weight Factor *C*_1_ for the Brillouin Function *S* = 5/2	Weight Factor *C*_2_ for the Brillouin Function *S* = 1/2	Linear Factor *k*, *T*/K
**1**	0.19	0.025	0	0.034
**2**	0.72	0.1	0	0.07
**3**	1.33	0.21	0.39	0.02

## Data Availability

The data presented in this study are available on request from the corresponding author.

## Supplementary Materials

The following are available online at https://www.mdpi.com/article/10.3390/ma14185445/s1: Figure S1: Mass spectrum of complex [Fe(L)_2_]NO_3_ (**1**). Similar MS spectra were obtained for other complexes; Figure S2: FTIR spectrum of complex [Fe(L)_2_]NO_3_ (**1**), KBr pellet; Figure S3: FTIR spectrum of complex [Fe(L)_2_]Cl (**2**), KBr pellet; Figure S4: FTIR spectrum of complex [Fe(L)_2_]PF_6_ (**3**), KBr pellet; Figure S5: Far-IR spectrum of complex [Fe(L)_2_]NO_3_ (**1**), CsBr pellet; Figure S6: Far-IR spectrum of complex [Fe(L)_2_]Cl (**2**), CsBr pellet; Figure S7: Far-IR spectrum of complex [Fe(L)_2_]PF_6_ (**3**), CsBr pellet; Figure S8: ^1^H NMR spectrum of complex [Fe(L)_2_]NO_3_ (**1**), CDCl_3_ solution; Figure S9: ^1^H NMR spectrum of complex [Fe(L)_2_]Cl (**2**), CDCl_3_ solution; Figure S10: ^1^H NMR spectrum of complex [Fe(L)_2_]PF_6_ (**3**), CDCl_3_ solution; Figure S11: TG, DSC curves (heating cycle) of complex **1**; Figure S12: TG and DSC curves (heating cycle) of complex **2**; Figure S13: The calculated absorption spectra for the *mer* isomer in the HS (*S* = 5/2) and LS (*S* = 1/2) states. The vertical lines showing the position of electronic transitions and their intensity (*f*—oscillator strength) were broadened by the Lorentz function; Figure S14: The calculated absorption spectra for the *fac* isomer in the HS (*S* = 5/2) and LS (*S* = 1/2) states. The vertical lines showing the position of electronic transitions and their intensity (*f*—oscillator strength) were broadened by the Lorentz function; Table S1: The calculated excitation energies (absorption wavelengths), oscillator strengths, natural transition orbital (NTO) pairs, and their eigenvalues (occupations) for selected excited states of the *mer* isomer (HS state); Table S2: The calculated excitation energies (absorption wavelengths), oscillator strengths, natural transition orbital (NTO) pairs, and their eigenvalues (occupations) for selected excited states of the *fac* isomer (HS state); Figure S15: Experimental diffraction patterns of complex [Fe(L)_2_]NO_3_ (**1**); Figure S16: Experimental diffraction patterns of complex [Fe(L)_2_]Cl (**2**); Figure S17: Experimental diffraction patterns of complex [Fe(L)_2_]PF_6_ (**3**).
