# Supplementary material for: Dendritic Iron(III) Carbazole Complexes: Structural, Optical, and Magnetic Characteristics
Source: Materials (Basel). 2021 Sep 20;14(18):5445. doi: 10.3390/ma14185445 (PMC8467834; doi:10.3390/ma14185445)
Supplement: Supplementary file 1 [file materials-14-05445-s001.zip › materials-1298267-supplementary.pdf]

SUPPLEMENTARY MATERIALS

Dendritic Iron(III) Carbazole Complexes: Structural, Optical, and Magnetic Characteristics

Matvey Gruzdev, Ulyana Chervonova, Arkadiy Kolker, Nadezhda Fomina, Ekaterina Zueva, Valerya Vorobeva, Denis Starichenko, and Alexander Korolev

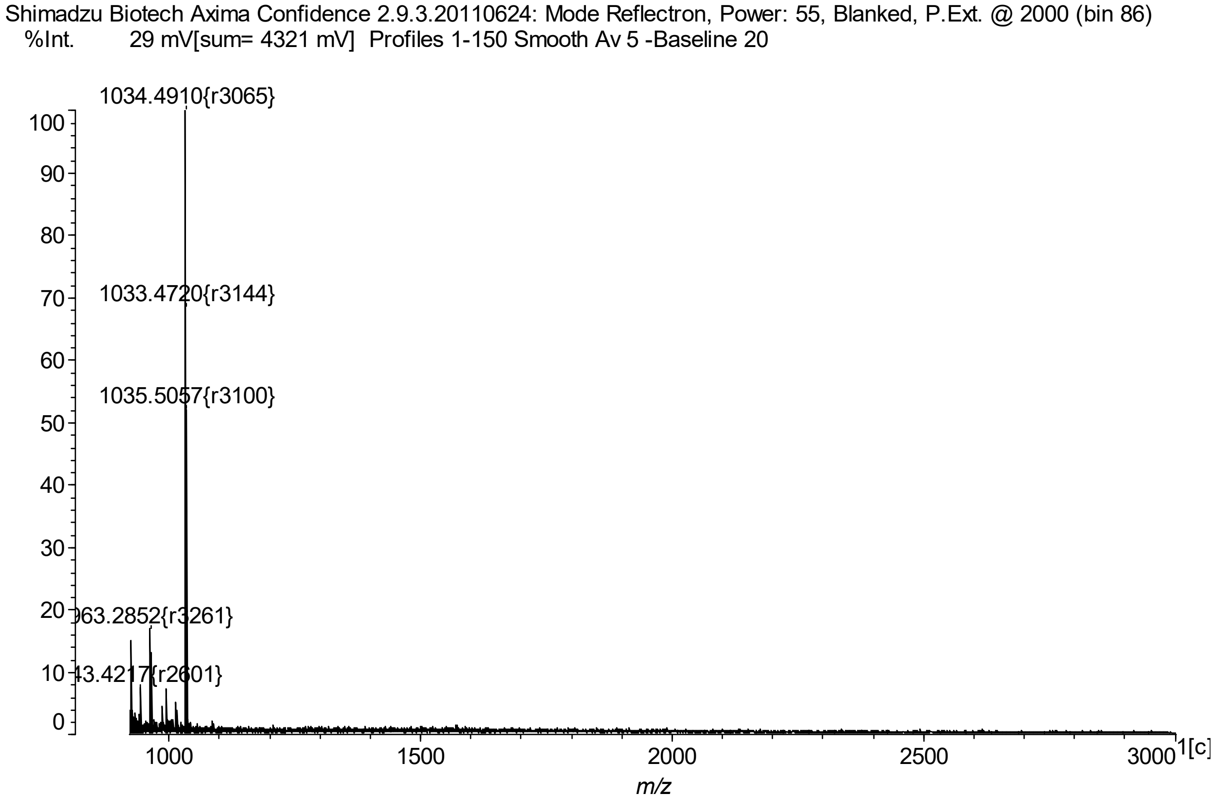

Figure S1. Mass spectrum of complex $[Fe(L)_2]NO_3$ (**1**). Similar MS spectra were obtained for other complexes.

This type of MS spectra is typical for all complexes. Chelated compounds with a high degree of branching do not give stable molecular ions, but are completely fragmented in the range of *m/z* values from 200 to 2300. The formation of a Schiff base is indicated by the presence of fragmented ions with an *m/z* value of ~ 1034 $[L]^+$. It was not possible to detect more intense stable molecular ions even when changing the matrix for MALDI-ToF analysis.

IR-spectra

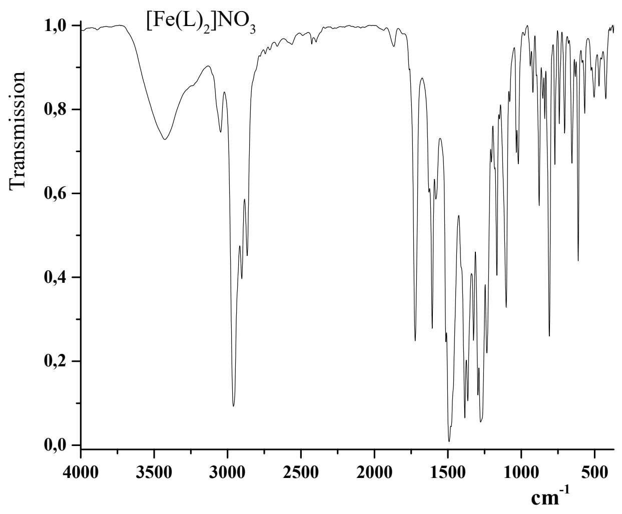

Figure S2. FT-IR spectrum of complex $[Fe(L)_2]NO_3$ (**1**), KBr pellet.

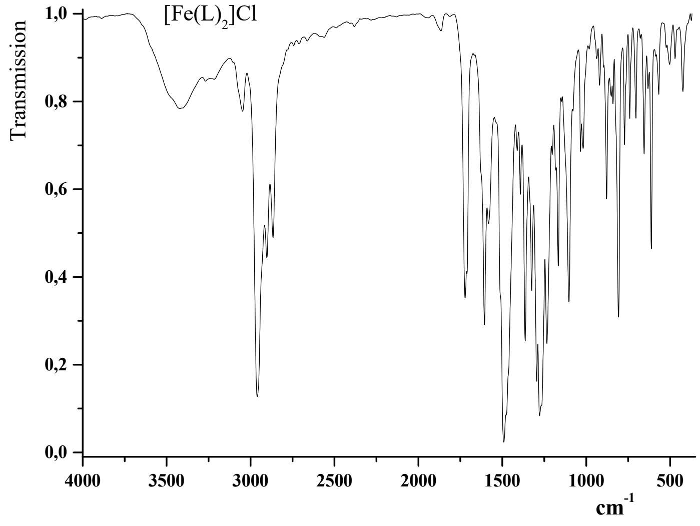

Figure S3. FT-IR spectrum of complex $[Fe(L)_2]Cl$ (**2**), KBr pellet.

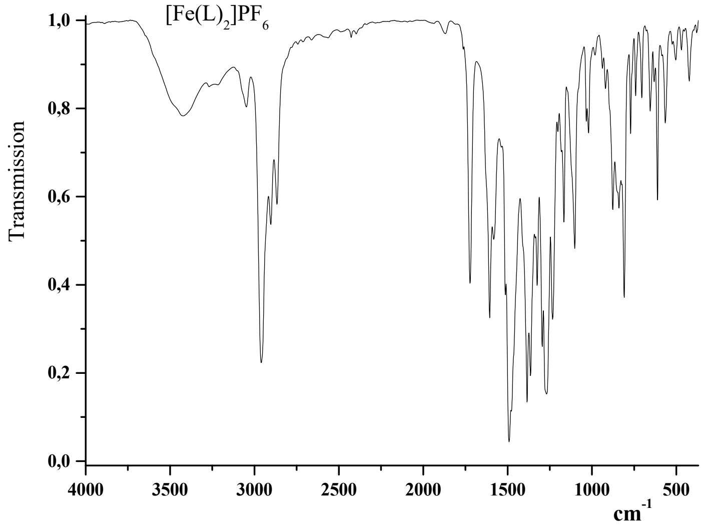

Figure S4. FT-IR spectrum of complex $[Fe(L)_2]PF_6$ (**3**), KBr pellet.

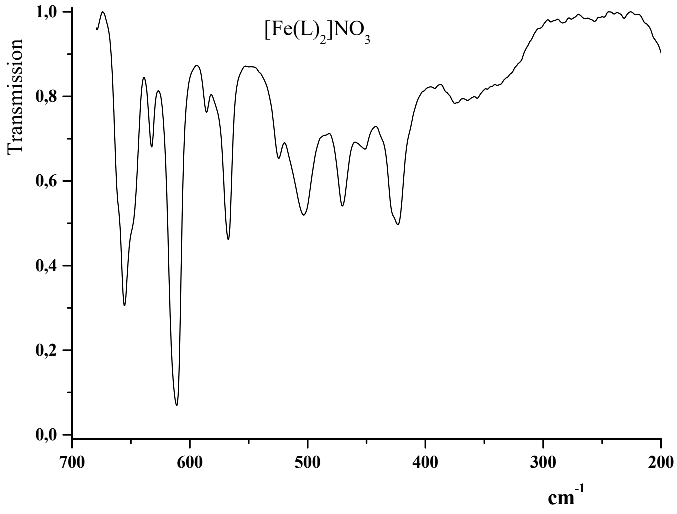

Figure S5. Far-IR spectrum of complex [Fe(L)$_2$]NO_3 (**1**), CsBr pellet.

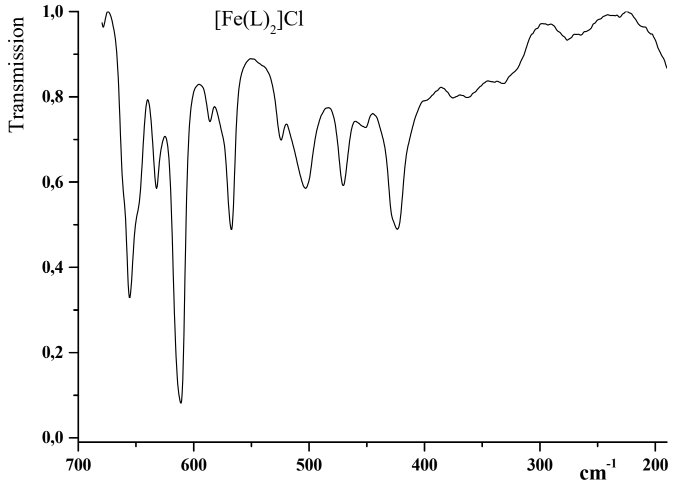

Figure S6. Far-IR spectrum of complex [Fe(L)$_2$]Cl (**2**), CsBr pellet.

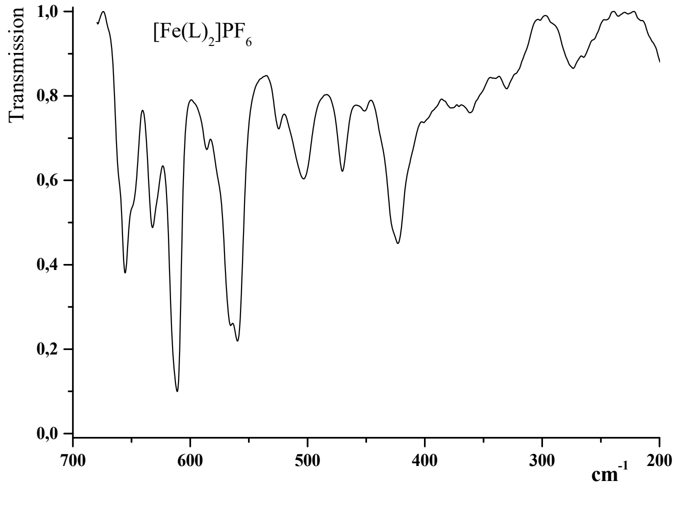

Figure S7. Far-IR spectrum of complex [Fe(L)$_2$]PF_6 (**3**), CsBr pellet.

^{1}H NMR spectra

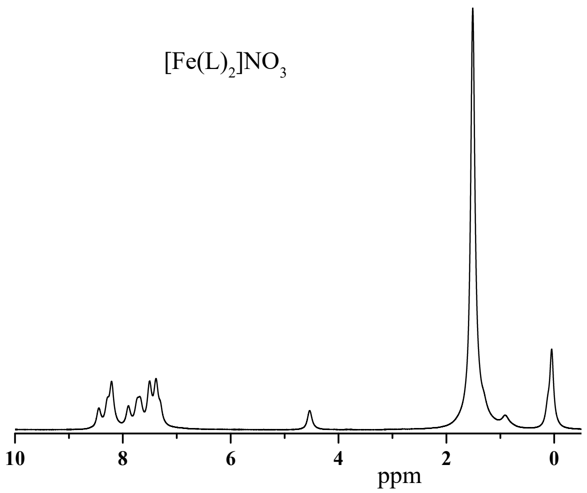

Figure S8. ^{1}H NMR spectrum of complex $[Fe(L)_2]NO_3$ (**1**), $CDCl_3$ solution.

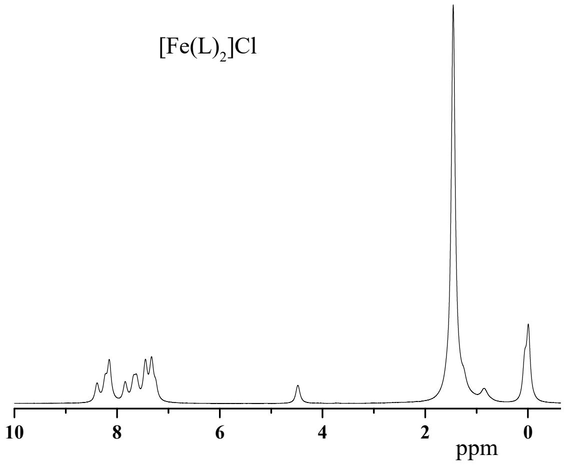

Figure S9. ^{1}H NMR spectrum of complex $[Fe(L)_2]Cl$ (**2**), $CDCl_3$ solution.

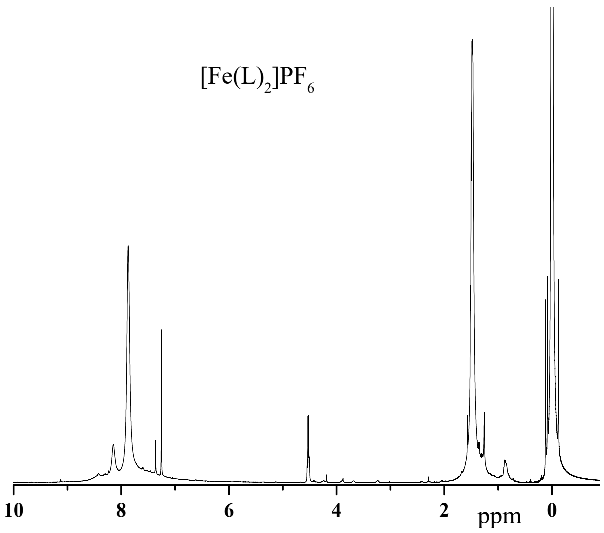

Figure S10. ^{1}H NMR spectrum of complex $[Fe(L)_2]PF_6$ (**3**), $CDCl_3$ solution.

DSC/TG curves

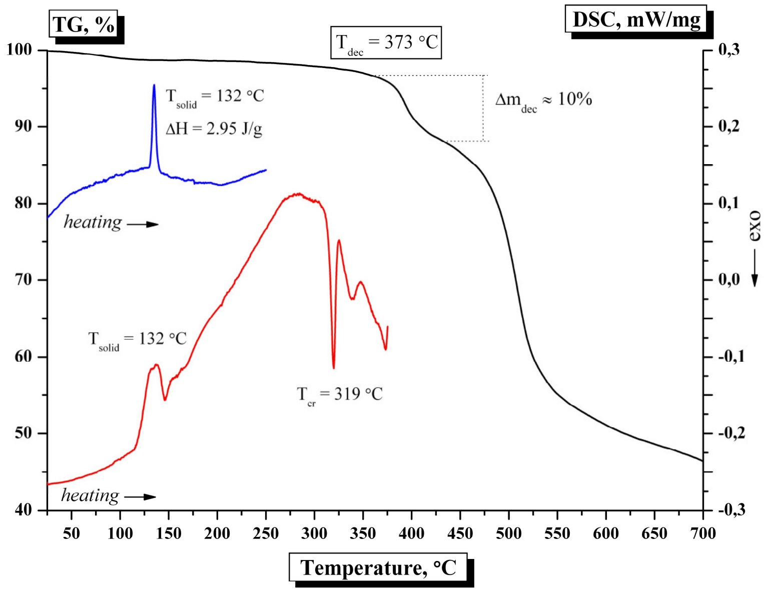

Figure S11. TG, DSC curves (heating cycle) of complex **1**.

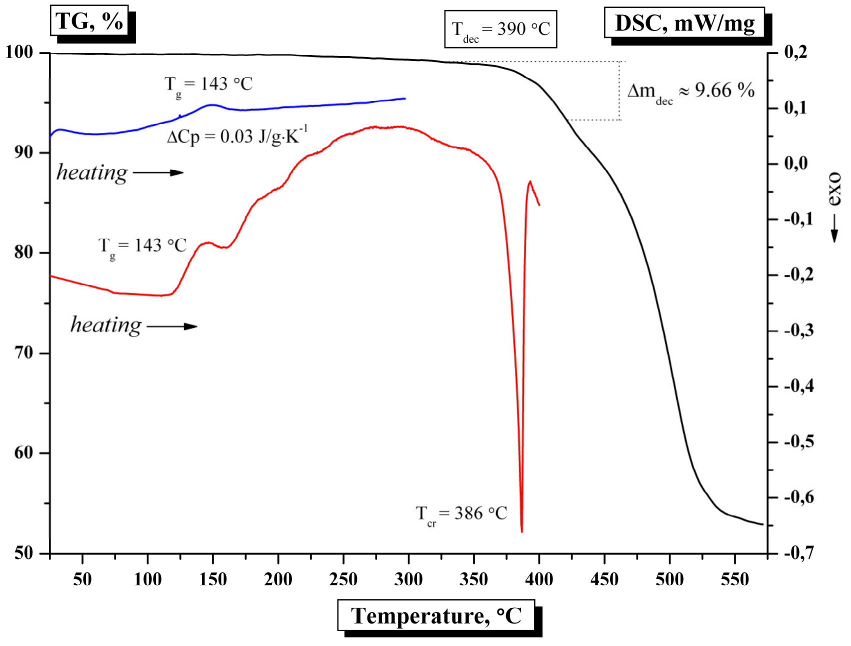

Figure S12. TG, DSC curves (heating cycle) of complex **2**.

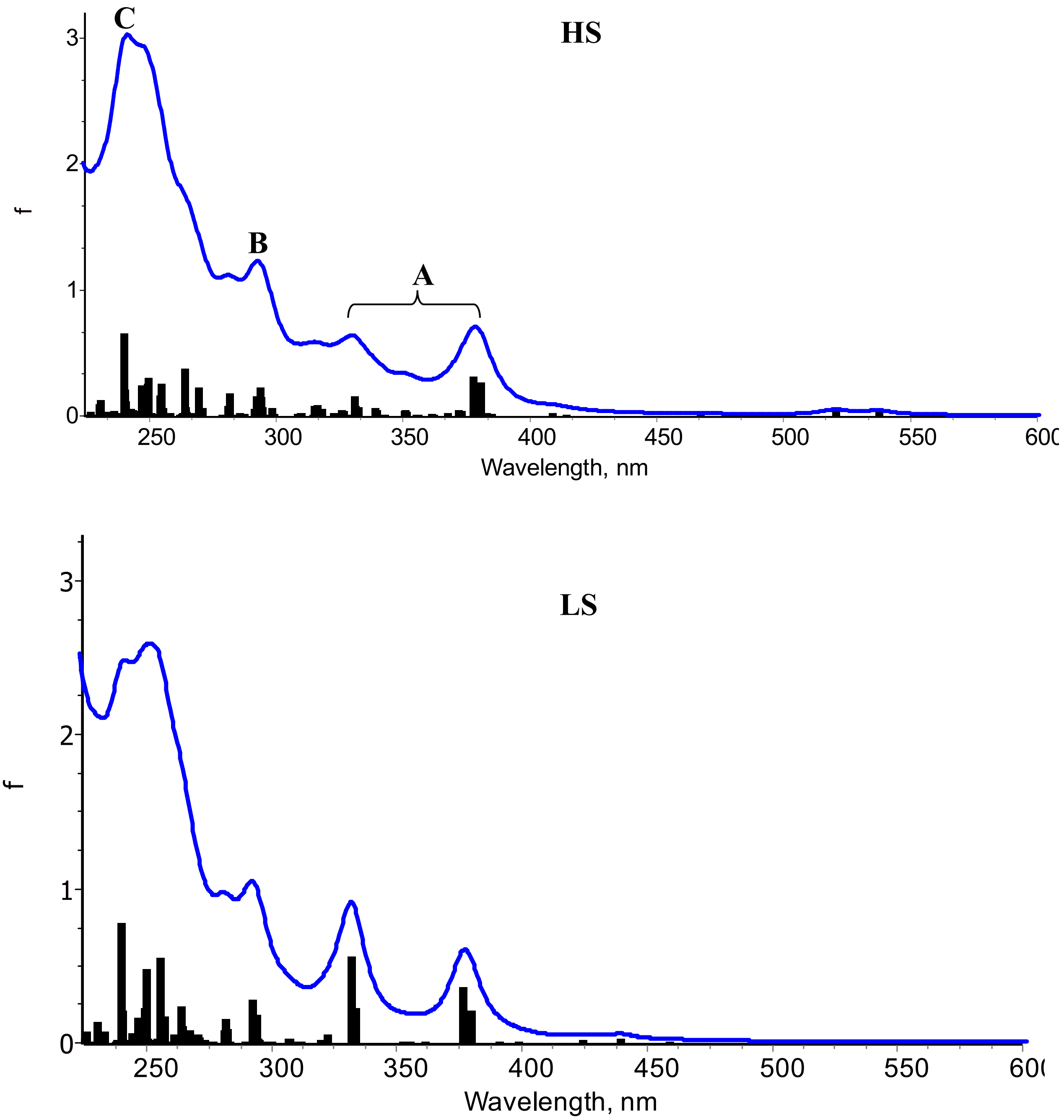

Figure S13. The calculated absorption spectra for the *mer* isomer in HS (S = 5/2) and LS (S = 1/2) states. The vertical lines showing the position of electronic transitions and their intensity (f – oscillator strength) were broadened by the Lorentz function.

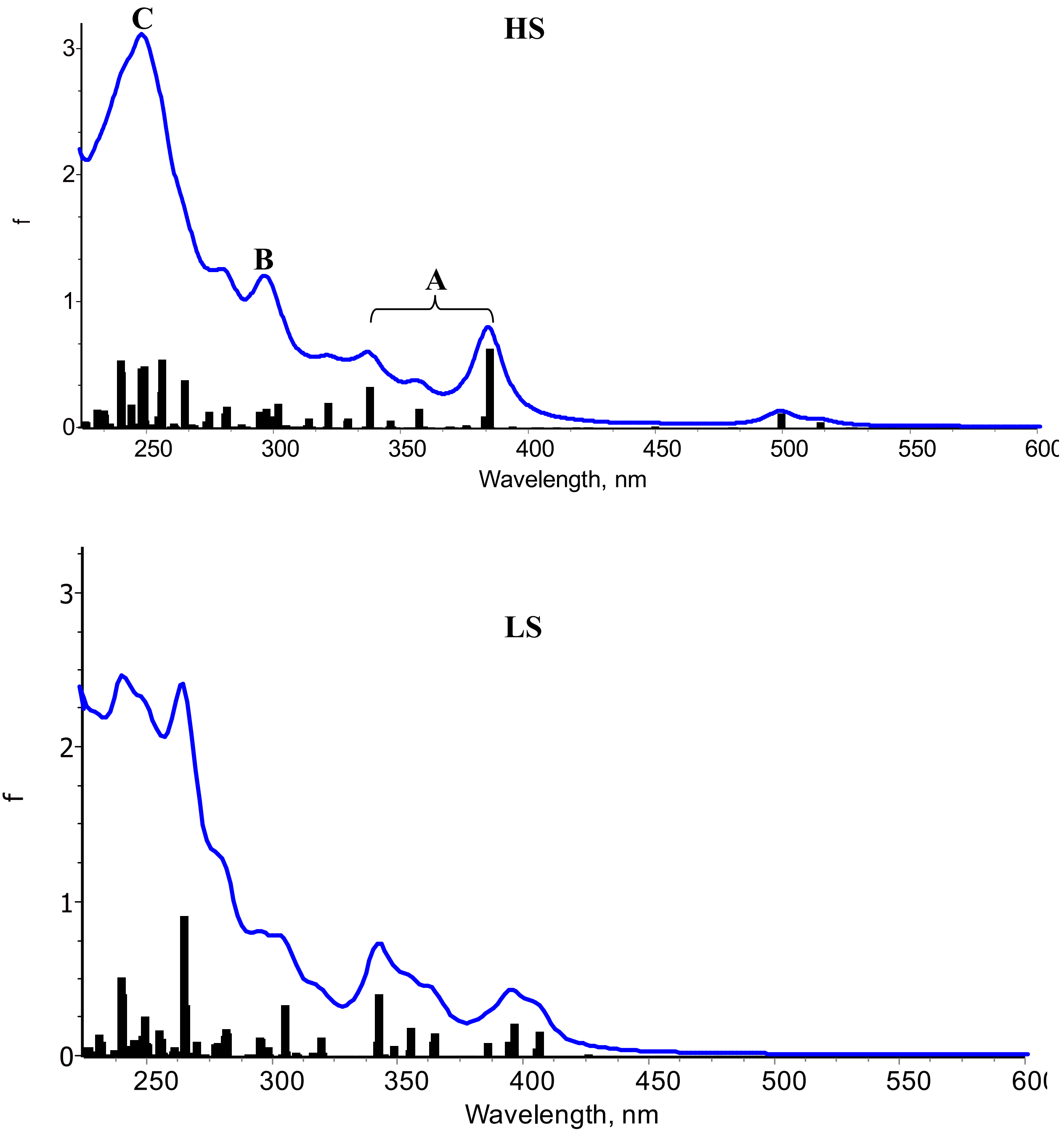

Figure S14. The calculated absorption spectra for the *fac* isomer in HS (S = 5/2) and LS (S = 1/2) states. The vertical lines showing the position of electronic transitions and their intensity (f – oscillator strength) were broadened by the Lorentz function.

Table S1. The calculated excitation energies (absorption wavelengths), oscillator strengths, natural transition orbital (NTO) pairs and their eigenvalues (occupations) for selected excited states of the *mer* isomer (HS state).

λ	f	Type	Assignment	Occ		NTO pair	
380	0.27	ILCT<br>LMCT	$\pi_{carb} \rightarrow \pi^*_{benz}$<br>$\pi_{carb} \rightarrow \pi^*_{sal}$	α	0.20	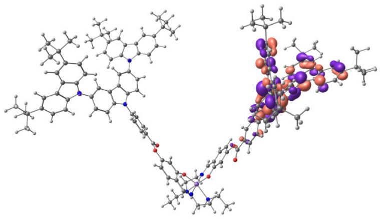	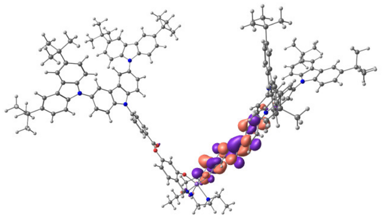
					0.17	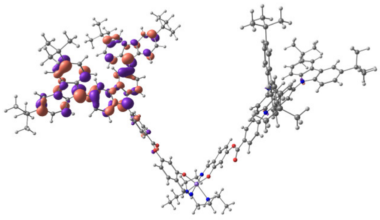	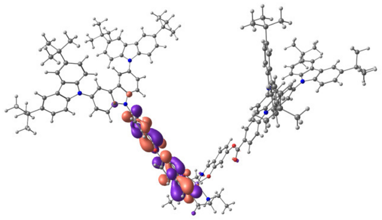
			$\pi_{carb} \rightarrow d_{Fe}$	β	0.24	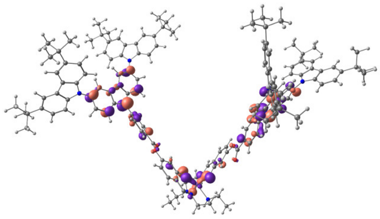	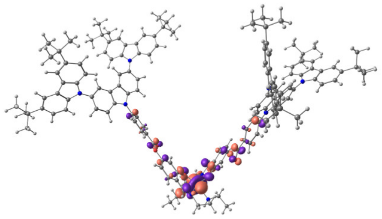
			$\pi_{carb} \rightarrow \pi^*_{benz}$<br>$\pi_{sal} \rightarrow d_{Fe}$		0.15	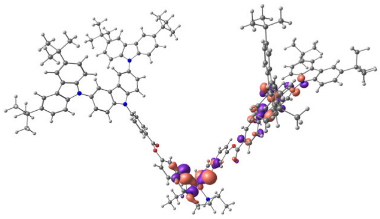	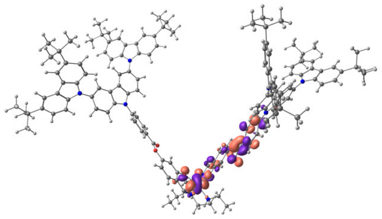
			$\pi_{carb} \rightarrow \pi^*_{benz}$<br>$\pi_{carb} \rightarrow d_{Fe}$		0.13	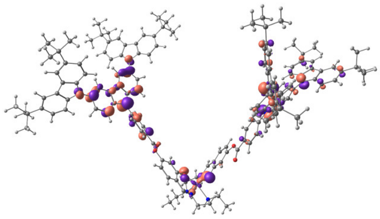	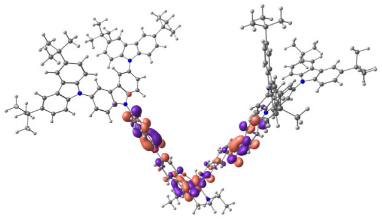
378	0.31	ILCT<br>LMCT	$\pi_{carb} \rightarrow \pi^*_{benz}$<br>$\pi_{carb} \rightarrow \pi^*_{sal}$	α	0.23	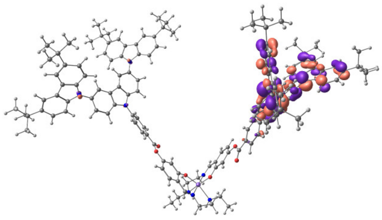	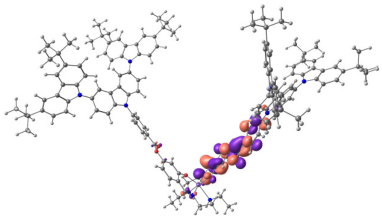
					0.19	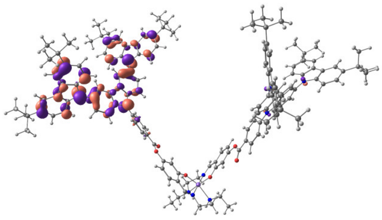	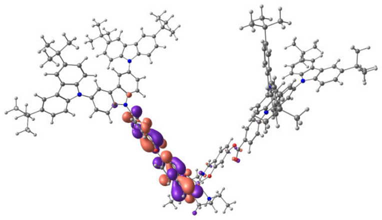

			$\pi_{carb} \to d_{Fe}$	β	0.32	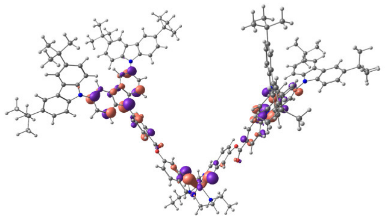	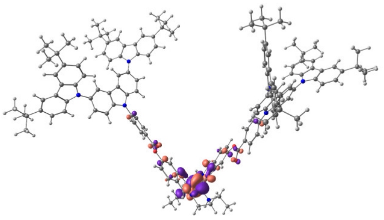
			$\pi_{carb} \to \pi^*_{benz}$<br>$\pi_{carb} \to d_{Fe}$		0.14	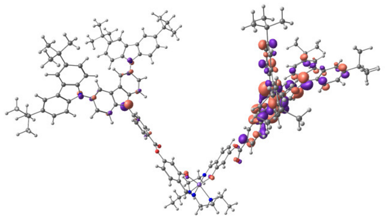	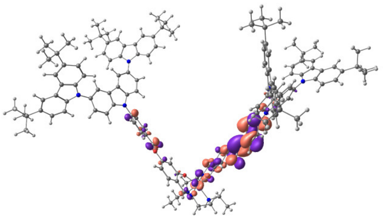
331	0.16	LMCT<br>ILCT	$\pi_{carb} \to \pi^*_{benz}$<br>$\pi_{carb} \to \pi^*_{sal}$	α	0.12	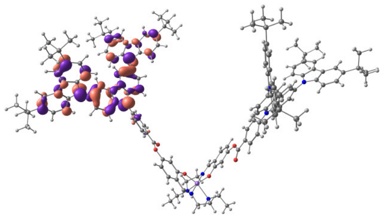	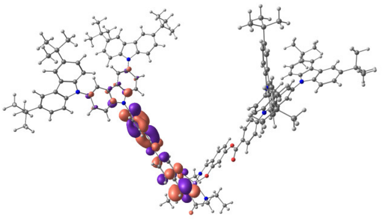
			$\pi_{carb} \to d_{Fe}$	β	0.54	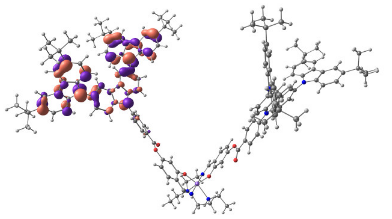	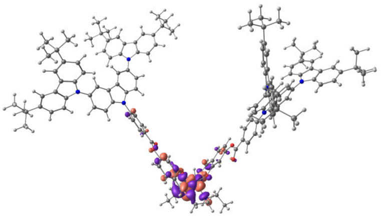
			$\pi_{sal} \to d_{Fe}$		0.16	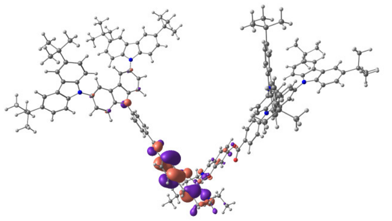	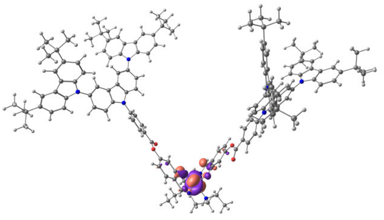

Table S2. The calculated excitation energies (absorption wavelengths), oscillator strengths, natural transition orbital (NTO) pairs and their eigenvalues (occupations) for selected excited states of the *fac* isomer (HS state).

λ	f	Type	Assignment	Occ		NTO pair	
385	0.63	ILCT	$\pi_{carb} \rightarrow \pi^*_{benz}$<br>$\pi_{carb} \rightarrow \pi^*_{sal}$	α<br>β	0.40<br>0.31	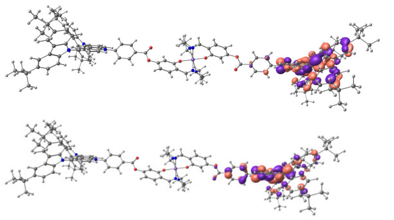	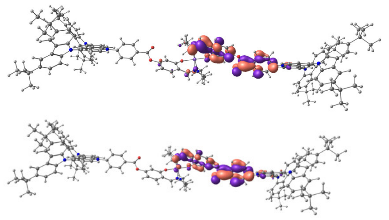
357	0.15	ILCT<br>LMCT	$\pi_{carb} \rightarrow \pi^*_{sal}$<br>$\pi_{carb} \rightarrow \pi^*_{sal}$<br>$\pi_{carb} \rightarrow d_{Fe}$	α<br>β	0.46<br>0.22	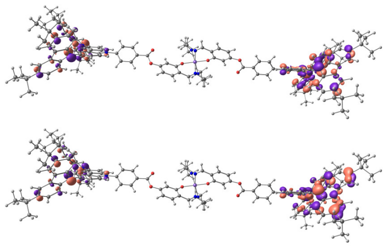	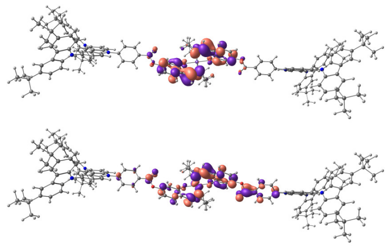
338	0.32	ILCT<br>LMCT	$\pi_{carb} \rightarrow \pi^*_{sal}$<br>$\pi_{carb} \rightarrow \pi^*_{sal}$<br>$\pi_{carb} \rightarrow d_{Fe}$<br>$\pi_{carb} \rightarrow \pi^*_{benz}$<br>$\pi_{sal} \rightarrow d_{Fe}$	α<br>β	0.15<br>0.46<br>0.20	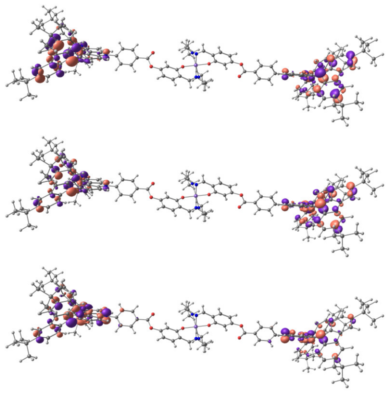	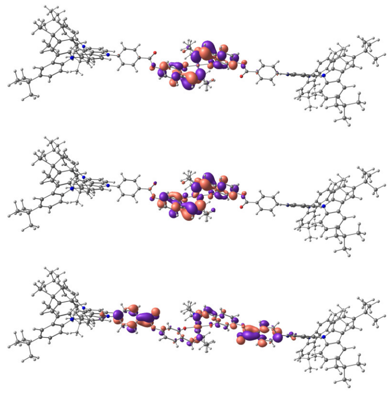

X-ray powder diffraction data

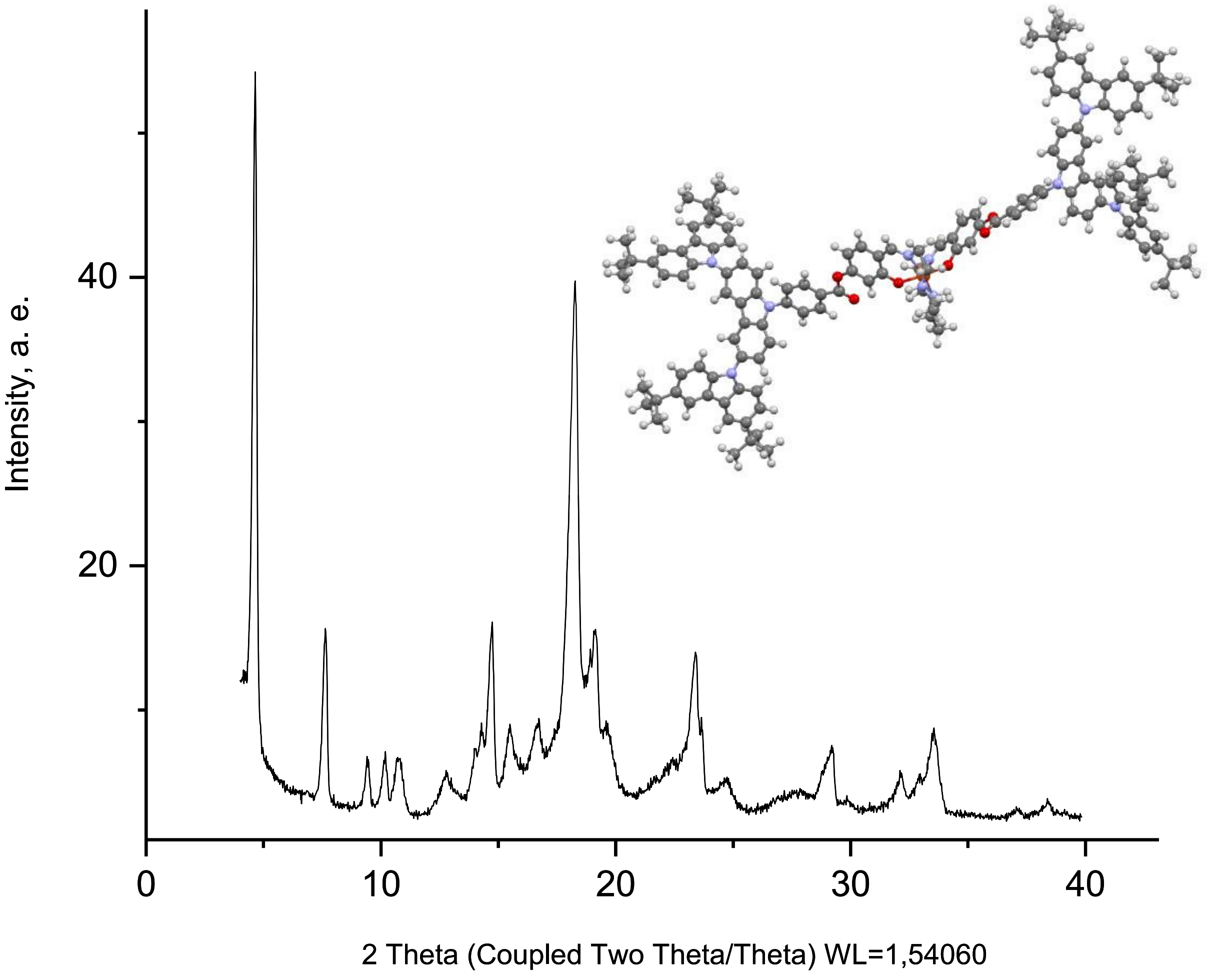

Figure S15. Experimental diffraction patterns of complex $[Fe(L)_2]NO_3$ (**1**).

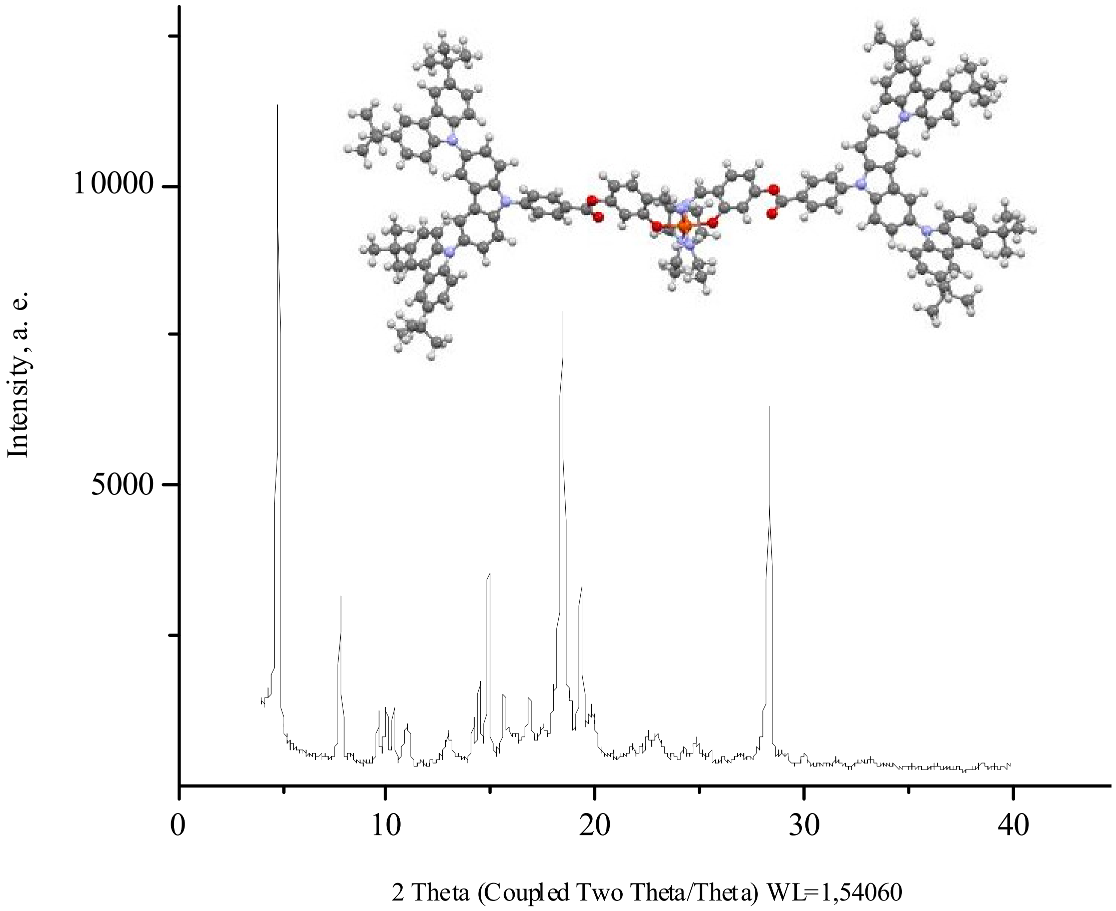

Figure S16. Experimental diffraction patterns of complex [Fe(L)$_2$]Cl (**2**).

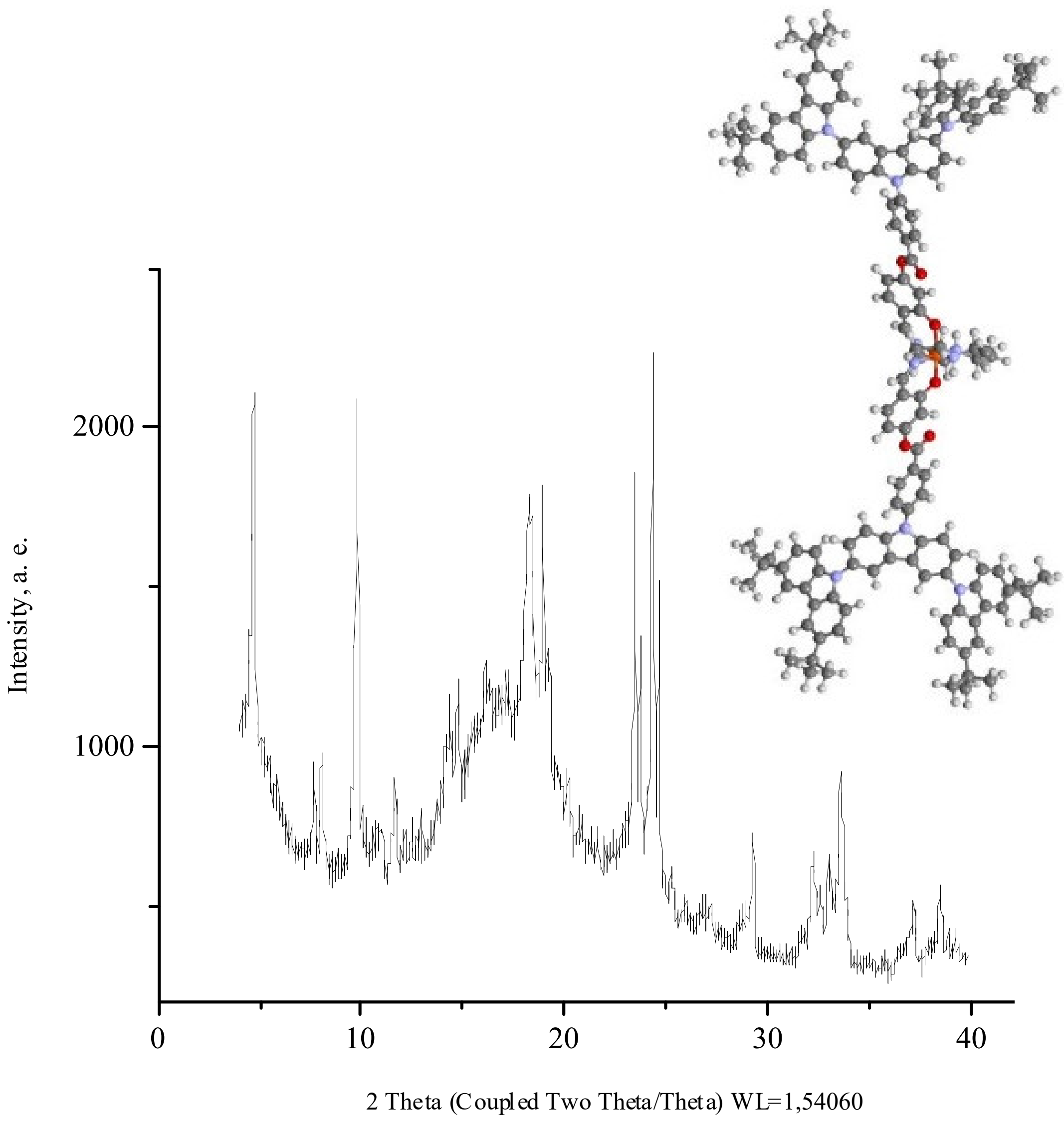

Figure S17. Experimental diffraction patterns of complex $[Fe(L)_2]PF_6$ (**3**).
